# A mobile pathogenicity chromosome in *Fusarium oxysporum* for infection of multiple cucurbit species

**DOI:** 10.1038/s41598-017-07995-y

**Published:** 2017-08-22

**Authors:** Peter van Dam, Like Fokkens, Yu Ayukawa, Michelle van der Gragt, Anneliek ter Horst, Balázs Brankovics, Petra M. Houterman, Tsutomu Arie, Martijn Rep

**Affiliations:** 1 Molecular Plant Pathology, Swammerdam Institute for Life Sciences, University of Amsterdam, Amsterdam, The Netherlands; 2 Laboratory of Plant Pathology, Graduate School of Agriculture, Tokyo University of Agriculture and Technology (TUAT), Fuchu, Tokyo Japan; 3 Westerdijk Fungal Biodiversity Institute, Utrecht, The Netherlands

## Abstract

The genome of *Fusarium oxysporum* (Fo) consists of a set of eleven ‘core’ chromosomes, shared by most strains and responsible for housekeeping, and one or several accessory chromosomes. We sequenced a strain of Fo f.sp. *radicis*-*cucumerinum* (Forc) using PacBio SMRT sequencing. All but one of the core chromosomes were assembled into single contigs, and a chromosome that shows all the hallmarks of a pathogenicity chromosome comprised two contigs. A central part of this chromosome contains all identified candidate effector genes, including homologs of *SIX6*, *SIX9*, *SIX11* and *SIX* *13*. We show that *SIX*6 contributes to virulence of Forc. Through horizontal chromosome transfer (HCT) to a non-pathogenic strain, we also show that the accessory chromosome containing the *SIX* gene homologs is indeed a pathogenicity chromosome for cucurbit infection. Conversely, complete loss of virulence was observed in Forc016 strains that lost this chromosome. We conclude that also a non-wilt-inducing Fo pathogen relies on effector proteins for successful infection and that the Forc pathogenicity chromosome contains all the information necessary for causing root rot of cucurbits. Three out of nine HCT strains investigated have undergone large-scale chromosome alterations, reflecting the remarkable plasticity of Fo genomes.

## Introduction

*Fusarium oxysporum* Schlechtend.: Fr. f.sp. *radicis*-*cucumerinum* Vakalounakis (Forc) is the causal agent of root and stem rot in cucurbits resulting in severe damage, particularly in greenhouse cucumber (*Cucumis sativus*) and muskmelon (*C*. *melo*). The disease was first described in Greece in 1989 by Vakalounakis, who identified the pathogen as a new *forma specialis* of *F*. *oxysporum* (Fo)^1^. Forc has since been recorded in several other countries including Canada, France, Spain, China, Turkey and Israel^2–6^. Unlike *Fusarium* wilt caused by Fo f.sp. *cucumerinum* (Foc) or Fo f.sp. *melonis* (Fom), the main symptoms caused by Forc are external rotting of the root and stem and profuse sporulation in the rotted tissue^1, 3^. Still, the infection mechanism appears to be the same: the fungus invades the roots and colonizes the xylem vessels of the plant (Video S1).

*Formae speciales* of Fo typically have a very narrow host range, often restricted to a single plant species^7, 8^. Forc is exceptional because its host range includes not only cucumber and melon, but also additional *Cucurbitaceae* species such as watermelon (*Citrullus lanatus*), squash (*Cucurbita pepo*) and gourd (*Luffa aegyptiaca*)^1, 3, 6^. The genetic mechanism underlying the difference in disease symptoms (root rot versus wilt) as well as the extended host range of this pathogen are unknown, but may be associated with the suite of effector genes present in the genome of this *forma specialis* ^9^. We found that Forc strains possess four Secreted In Xylem (*SIX*) gene homologs: *SIX6*, *SIX9*, *SIX*11 and *SIX*13, which encode small secreted proteins originally identified in tomato-infecting strains^10–12^. Additionally, we found several other genes encoding candidate effectors based on small size, predicted secretion signal, and vicinity to a “miniature impala” (mimp) transposable element, including a secreted astacin-like metalloprotease^9^.

The genome of Fo is typically divided into a set of eleven ‘core’ chromosomes, with sequences generally conserved in all *Fusarium* species, and responsible for housekeeping, and one or several transposon-rich and gene-poor ‘accessory’ chromosomes^13^. In Fo f.sp. *lycopersici* (Fol), one of these accessory chromosomes was shown to be required for pathogenicity towards tomato^14^. Moreover, it can be horizontally transferred to the non-pathogenic strain Fo47, thereby transforming this strain into a tomato pathogen^15, 16^. In *de novo* Illumina assemblies, accessory chromosomes are typically dispersed over many contigs or scaffolds due to their high repeat-content, making it impossible to determine how many accessory chromosomes are present in a strain. Three Forc strains have been sequenced so far, each resulting in assemblies of several hundred scaffolds^9^. A solution to the high level of fragmentation of Fo assemblies could be long-read sequencing technology, such as PacBio Single Molecule Real-Time (SMRT) sequencing. This would allow the multiple kb-sized repetitive elements to be spanned by individual reads, leading to much larger contigs.

The aims of this study were to (i) determine the genome structure of Forc, (ii) investigate whether Forc, like wilt-causing strains of Fo, relies on effector proteins for successful colonization and (iii) identify which part(s) of the Forc genome are necessary for the root- and shoot-rot phenotype as well as the extended host range of Forc. To reach these aims, we applied SMRT sequencing of a representative strain of Forc (strain Forc016) as well as Fom (Fom001; NRRL26406) as a step towards answering the question what differentiates Forc from strains causing wilt.

## Results

### A corrected SMRT assembly of Forc contains 33 sequences including 12 (near) full-length chromosomes

In order to obtain a better understanding of the genome composition of Forc, an HGAP.3 *de novo* assembly was generated for Forc016, a strain previously sequenced by Illumina^9^. The initial SMRT assembly consisted of 41 contigs, including seven contigs that contained ribosomal DNA (rDNA) repeats. Two of these show rDNA sequences at one end and telomeric repeats (CCCTAA) on the other end, indicating that they together form chromosome 2^13^. The rDNA copy number was estimated through Illumina read coverage (~98 copies), and the two contigs were joined to reconstruct chromosome 2 (N.B. numbering of core chromosomes follows the Fol4287 reference genome). Three rDNA repeats of each contig were kept. The 91 copies in between were filled with the first rDNA repeat of the first contig.

Chromosome 13 was also assembled into two contigs, but an overlap of 13,396 nucleotides and synteny to the SMRT assemblies of Fom001 (Fig. S1), as well as a related *Fusarium* species, *F*. *subglutinans*, were found (B. Brankovics, personal communication). This allowed us to merge these sequences into chromosome 13.

**Figure 1:**
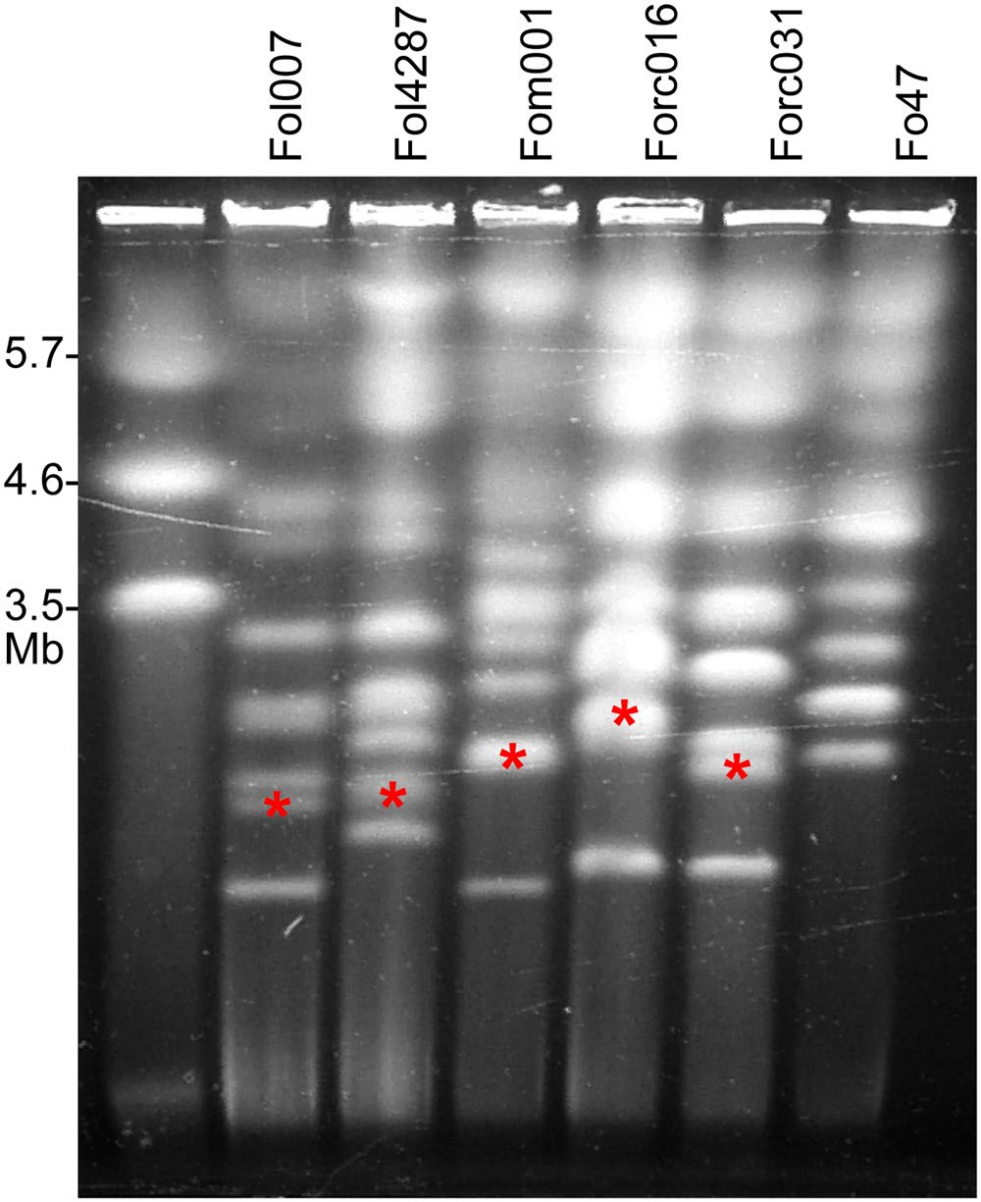
Electrophoretic karyotypes of strains belonging to *F*. *oxysporum* f.sp. *lycopersici* (Fol007, Fol4287), *melonis* (Fom001) or *radicis*-*cucumerinum* (Forc016, Forc031) and non-pathogenic strain Fo47. A red asterisk indicates the location of the radioactive Fol-*SIX6* probe hybridization signal, identifying the chromosomes potentially involved in pathogenicity. Fo47, a non-pathogenic strain, does not have a *SIX6* homolog. The left lane shows the karyotype of *Schizosaccharomyces pombe*, applied as a marker. This image is cropped, the original gel photograph can be found in Supplementary Fig. S11.

Contour-clamped homogeneous electric field (CHEF) electrophoretic karyotyping followed by Southern blotting and hybridization with a radioactive probe generated from a Fol-*SIX*6 Polymerase Chain Reaction (PCR) product revealed that the *SIX6* sequence is present on a ~2.5 megabase (Mb)-sized chromosome in Forc016 (Fig. 1, Fig. S2). This chromosome is present in the SMRT assembly as two separate contigs (13 and 17) of which the ends have an overlap of 586 nucleotides. Comparison to the Fom001 SMRT assembly revealed that this chromosome is largely syntenic to contig 127 of Fom001, but with a large (1.448 Mb) inversion between inverted, highly similar regions of about 200 kb (Fig. S3). Either end of this region matched the end of Forc016 contig 17. However, when the 1.448 Mb region was manually inverted, not a single nucleotide polymorphism (SNP) was found in the pairwise sequence alignment, whereas in the original assembly three single nucleotide InDel mismatches were identified (data not shown). We therefore conclude that it is more likely that the 1.448 Mb region is in the reverse orientation and we adjusted this manually. One contig containing the mitochondrial DNA (mtDNA) sequence was identified by BLAST, removed from the SMRT assembly and the 47,541 nucleotide-long (annotated) mitogenome generated through Illumina reads by the GRAbB program^17^ was added (Brankovics *et al*., submitted).

The final, manually corrected assembly of Forc016 is composed of eleven core chromosomes, one pathogenicity chromosome, twenty unpositioned sequences with a cumulative size of 2.572 Mb and the mitochondrial genome (Table 1). Nine of the unpositioned sequences end in telomeric repeats, indicating that they should probably be attached to the ends of chromosome-sized contigs that lack a telomere. The assembly is of a very high quality, with the L90 being reached with only eleven sequences.

**Table 1:** Comparison of the Forc016 genome assembly generated with Illumina HiSeq 2500 reads with the manually corrected SMRT HGAP.3 assembly and the raw SMRT HGAP.3 assembly of Fom001.

	Forc016 Illumina HiSeq	Forc016 SMRT	Fom001 SMRT
Assembly size	50,061,337	52,860,752	60,704,002
Ambiguous bases (Ns)	85,353	0	0
Gaps	902	0	0
GC (%)	47.63	47.69	47.65
Mean sequence length^a^	59,244	1,651,898	632,333
Shortest sequence length^a^	505	4587	5,714
Longest sequence length^a^	2,409,929	6,470,671	6,402,286
L50 (kbp)	575.0 (n = 21)	4,490.1 (n = 5)	4,357.5 (n = 6)
L70 (kbp)	237.5 (n = 49)	3,661.0 (n = 8)	2,962.0 (n = 10)
L90 (kbp)	37.7 (n = 149)	2,466.0 (n = 11)	761.3 (n = 17)
Coverage	91 X	72 X	59 X
# Sequences^a^	845	32 + mtDNA	96
# Sequences having telomeric repeats on both ends^a^	0	5	0
# Sequences having telomeric repeats on one end^a^	0	15	19
# Sequences having no telomeric repeats^a^	845	12	77

^a^‘Sequences’ refers to scaffolds (Illumina assembly) or contigs (SMRT assembly).

Two large sequence duplications are present on contig 53 of the SMRT assembly (Fig. S4E). Because of its size, this contig is likely a large part of one of the small (±1–1.5 Mb) accessory chromosome shown in Fig. 1. This is supported by the fact that it contains a GC-content drop typical of a centromeric region (Fig. S4B).

**Figure 2:**
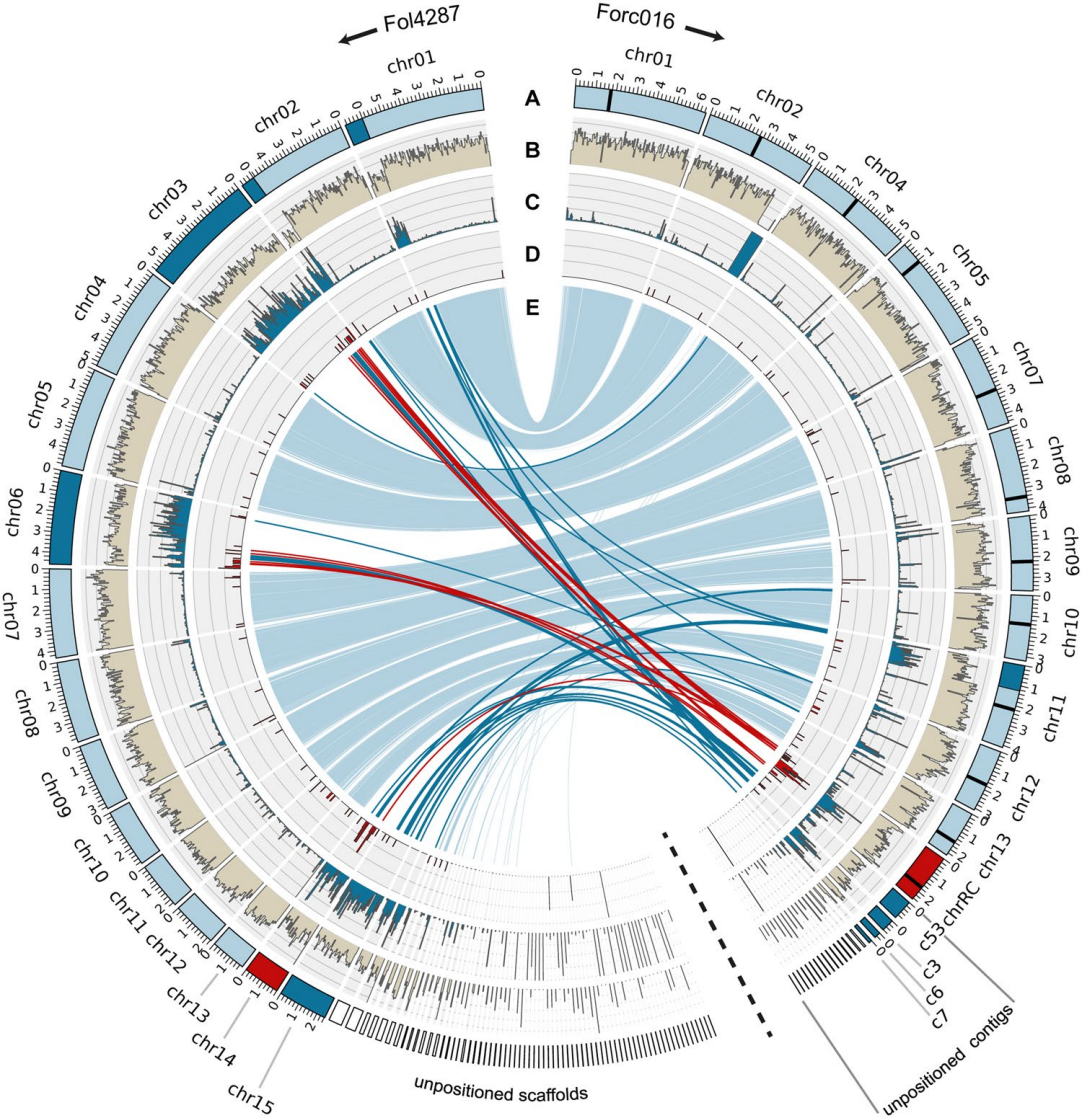
The SMRT genome assembly of Forc016 includes eleven core chromosomes, several repeat-rich, gene-poor accessory regions and one chromosome enriched in candidate effector genes. Comparison of the Forc016 assembly to that of Fol4287 reveals (**A**) eleven conserved core chromosomes (light blue), one putative pathogenicity chromosome (red) and several other accessory sequences (dark blue). Accessory regions typically have (**B**) low gene density and (**C**) high repeat density, both calculated here in 50 kb windows. The putative pathogenicity chromosome is marked by (**D**) the presence of many candidate effector genes. (**E**) Indicates nucmer alignments with the Fol4287 reference assembly: in red alignments to the putative pathogenicity chromosome of Forc016, in dark blue alignments from known accessory regions in Fol4287 (chr1B; chr2B; chr3; chr6; chr14; chr15) and in light blue the remaining alignments, mostly between core regions in both genomes.

Comparison of the Forc016 SMRT assembly to that of Fol4287, the reference genome of *F*. *oxysporum*, revealed that (i) the eleven core chromosomes are highly syntenic between the strains (with 98.9% sequence identity), (ii) the Forc016 assembly has six contigs that contain sequences that align to known Fol accessory regions – likely due to the presence of similar transposable elements (TEs) in both and (iii) one of these six contigs is a putative pathogenicity chromosome on which the *SIX6* sequence was identified earlier (Fig. 1) with a high number of repeats and effector candidates, that we named chr^RC^ (Fig. 2). Gene ontology (GO) terms related to metabolism, protein ADP-ribosylation and DNA integrity were found to be overrepresented among the predicted genes on chr^RC^ (Fig. S5, Table S1). We further focused on this chromosome.

### Most candidate effector genes reside in a subregion of chr^RC^

**Figure 3:**
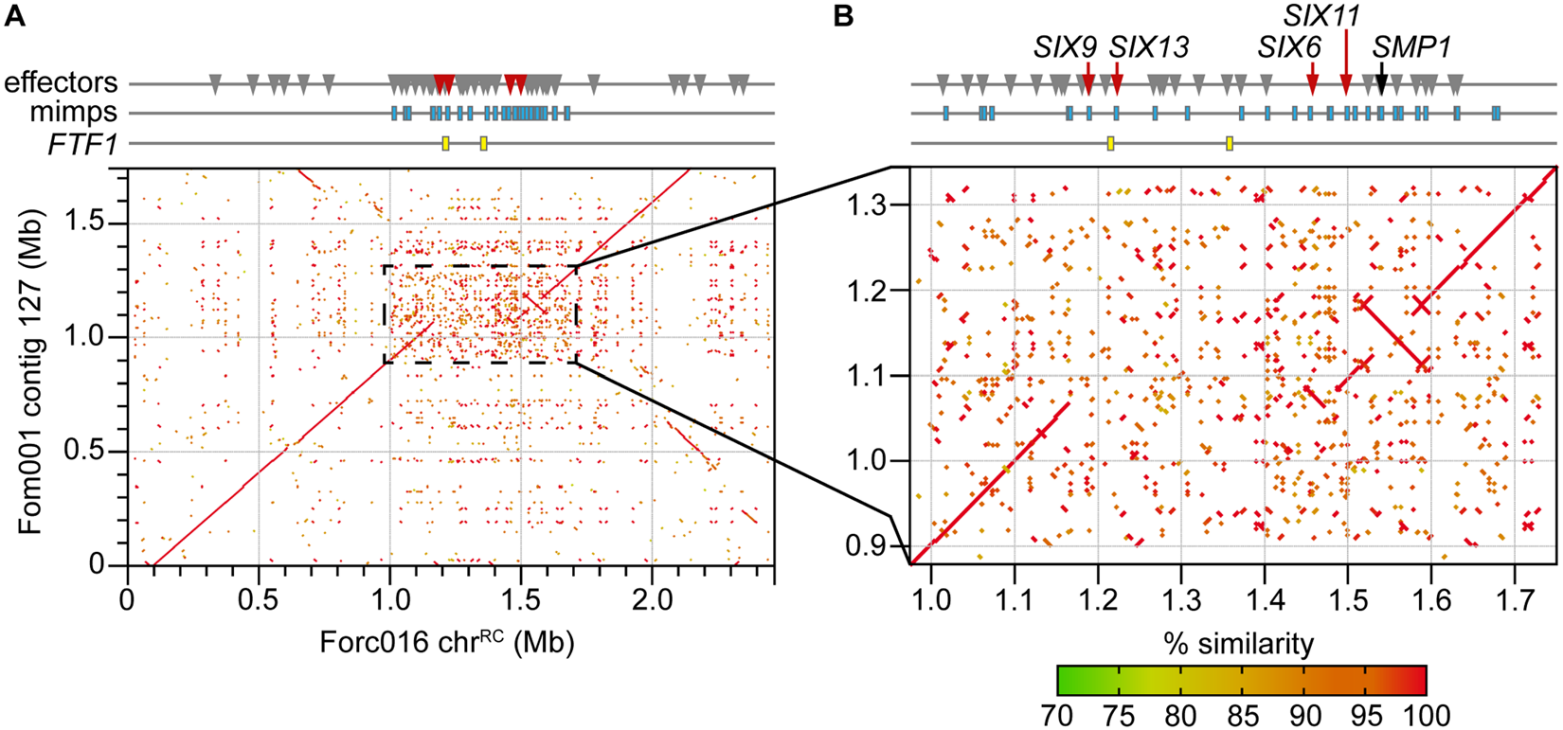
Comparison of chr^RC^ with Fom001 contig 127 reveals a highly dynamic central region containing the majority of the miniature impala (mimp) TEs and candidate effector genes of the Forc genome. (**A**) The chromosomes of the Fom and Forc strains are mostly syntenic, with large stretches showing 100% identity in this nucmer alignment. (**B**) The dynamic central region of the chromosome, about 700 kb in size, has the highest repeat density, 30 of the 35 mimps of the Forc genome and the majority of the Forc candidate effector genes, including *SIX9*, *13*, *6* and *11* and *SMP1*. Additionally, the two *FTF1* homologs present in the Forc genome are found here.

The putative pathogenicity chromosome of Forc016, chr^RC^, is highly similar (99.8%) to sequences present in the two other previously sequenced Forc strains^9^. Surprisingly, high similarity (>99%) was also observed with sequences in the genomes of Fom001, Fom004, Fom005, Fom006, Fom012, Fom13, Fom016 but not Fom009, Fom010, Fom011 (data not shown). A *de novo* HGAP.3 assembly for Fom001 was generated and we found that synteny is mostly preserved between chr^RC^ and Fom001 contig 127 (Fig. 3A). A notable exception is a central, ~700 kb region of the chromosome, which is exceptionally repeat-rich and of which ~300 kb is absent in Fom001 contig 127, flanked by several multi-kb inversions (Fig. 3B). Within the 700 kb region, 195 genes were predicted in the Forc016 SMRT assembly. 185 of these have a highly similar homolog in Fom001 (average nucleotide sequence identity is 99.1%). 135 of these genes (including *SIX6* and *SIX*11) are almost identical between the two strains, returning a BLAST hit percentage of 99.8% or higher, suggesting that they have been reshuffled recently. Only ten genes could not be identified with BLASTN (e-value <1e-20; perc_identity >90%; query coverage >70%) in Fom001: *SIX9* (g15834), three beta-lactamases (g15883, g15832, g15833), three hypothetical proteins (g15854, g15957, g15835), a cytochrome p450 (g15902), a putative lysine decarboxylase (g15903) and an NADH-flavin oxidoreductase (g15826). One or several of these genes may contribute to the ability of Forc to cause root rot in several cucurbit species.

Interestingly, this is exactly the region where 30 of the 35 full-size miniature impala (mimp) elements were found in the Forc genome. Two other mimps were found on chr12, one on chr11 and two more on contig 14, a contig of only 22.4 kb. Mimps are contextually associated with effector genes in *F*. *oxysporum* ^10, 18^. Indeed, the majority (51 out of 98) of candidate effector genes identified by BLAST from the list of 104 candidates that we identified earlier^9^ are localized in this region (Fig. 3B). Among these are the four *SIX* homologs that are present in Forc: *SIX6* (g15909), *SIX9* (g15834), *SIX11* (g16807) and *SIX13* (g15844). These were previously shown to be expressed during plant infection^9^. Additionally, two homologs of the *FTF1* transcription factor, associated with effector gene expression^19, 20^, are found here (g15884 and g15843).

In Fom001, homologs of *SIX1* (contig 22), *SIX6* (contig 127), *SIX11* (two copies; contig 10 and 127) and *SIX13* (contig 10) are present. Fom contigs 10 and 22 are 2.962 and 1.268 Mb in size, respectively, suggesting that Fom001 may have more than one chromosome associated with pathogenicity. The sequences of *SIX6* and *SIX11* that are located on Fom contig 127 are identical between Forc016 and Fom001. From the list of candidate Fom effectors in Schmidt *et al*.^18^, only candidate 1 A is present in the Forc016 assembly on chr^RC^ (100% identical between Fom and Forc). None of the other candidates, including *AVRFOM2*, is present in Forc.

### Six6 contributes to virulence of Forc

**Figure 4:**
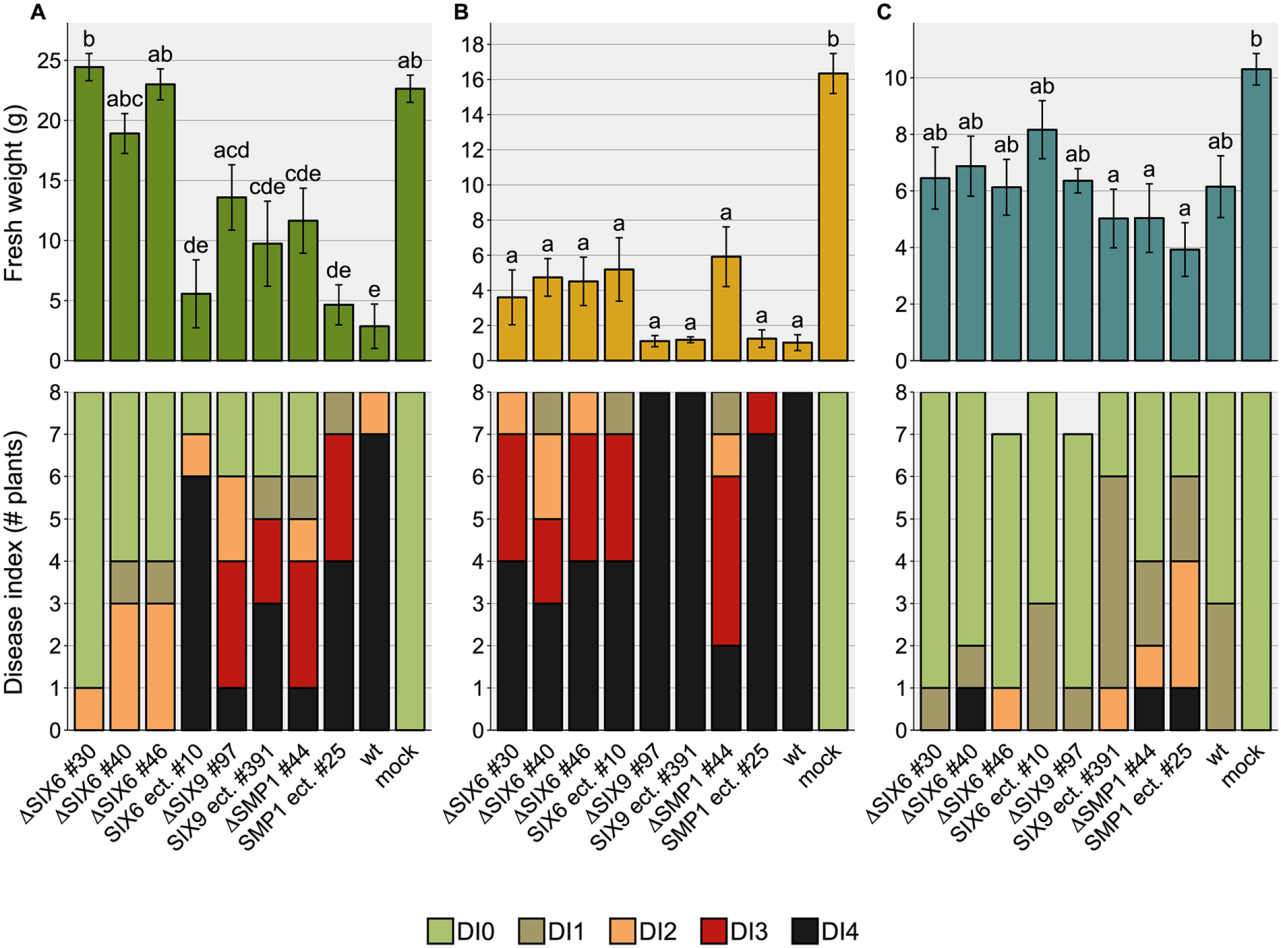
Three independent *SIX6* deletion strains cause less symptom development in cucumber. Fresh weight (±S.E.) and disease index (DI) of (**A**) cucumber, (**B**) melon and (**C**) watermelon plants were scored 14 days post inoculation. An ANOVA followed by a Tukey HSD test (p < 0.05) was performed to determine significance of the differences in the fresh weight measurements (significance categories shown with letters above the bars). Under the tested conditions (10^6^ spores/ml, 25 °C), three independent *SIX6* deletion strains caused reduced symptoms in cucumber compared to wildtype (wt) and an ectopic transformant (*SIX6* ect. #10). Knockout of the two other candidate virulence genes *SIX9* and *SMP1* did not have a significant effect on virulence. Under these conditions (25 °C), Forc causes only mild symptoms on watermelon.

In order to investigate the role candidate effectors play in Forc pathogenicity, knockout strains were generated through homologous recombination with a hygromycin resistance marker. Although this process is very inefficient in regions with many repeats, such as the region depicted in Fig. 4B, successful knockout was achieved for *SIX6*, *SIX9* and an astacin-like Secreted MetalloProtease gene (which we named *SMP1* (g15931); Fig. 3). All three genes are single copy in the Forc016 genome. Bioassays were conducted with cucumber, melon and watermelon plants to evaluate whether the fungus had become less pathogenic to one or several host plants upon loss of these genes.

When inoculated with 10^6^ spores/ml at an ambient temperature of 25 °C, the three independent *SIX6* knockout strains caused reduced disease symptoms in cucumber compared to a transformant with an ectopic integration of the T-DNA, as well as to the wild type Forc016 strain and the two other knockout strains (Fig. 4; pictures shown in Fig. S6). When tested at a lower ambient temperature of 18–20 °C, all strains caused quick and uniform death to all plants of the three tested species, indicating that these conditions are more favourable for Forc disease development and do not require Six6 (data not shown). Disruption of *SIX9* or *SMP1* did not significantly affect virulence under the tested conditions (Fig. 4). The absence of *SIX9* in Fom001 is therefore not responsible for the phenotypic difference between Fom and Forc.

### Cell wall degrading enzymes in rot symptom development

In a comparison between Forc and Foc strains, it was found that isolates of Forc have more pectolytic enzyme activity^6^. This may, in part, account for the crown rot and tissue maceration seen in root and shoot rot disease caused by Forc. Production of cell wall degrading enzymes (CWDEs) by Fo is well documented (reviewed in refs 21 and 22) and was shown to be positively correlated with virulence in Fo f.sp. *dianthi* ^23^. Individual knockout of CWDE- or protease-encoding genes, however, usually does not result in a detectable reduction in virulence in Fo^21, 22^. Site-directed mutagenesis of three amino acid residues located at the putative active site of an endopolygalacturonase from *F*. *verticillioides* (formerly *F*. *moniliforme*) did result in reduced macerating activity on potato medullary tissues^24^. This led Reignault *et al*. to hypothesize that pectinases are important for necrosis and maceration (e.g. by Forc), but are less important for vascular wilt disease^25^.

In total, 179 gene products are predicted to have proteolytic activity (ontology term GO:0006508) in the Forc genome, of which four are encoded on chr^RC^. Two of these possess a predicted signal peptide: *SMP1* and a subtilase gene. Polygalacturonase activity (GO:0004650) was predicted for 11 genes, none of which resides on chr^RC^. Likewise, none of six pectinesterase-encoding genes in the Forc genome (GO:0030599) resides on chr^RC^. Since knockout of *SMP1* did not result in reduced virulence, there may be functional redundancy with other proteases. Despite these observations, protease or CWDE activity may still be important during plant colonization and rot symptom development.

### Chr^RC^ is a mobile chromosome

Chr^RC^ is similar to the mobile Fol pathogenicity chromosome^15^ in that it is repeat-rich, gene-poor and contains most candidate effector genes, of which at least one (*SIX6*) contributes to virulence towards cucumber. In order to assess whether this chromosome could be horizontally transferred to other strains, a co-cultivation experiment was performed. Forc016*∆SIX6*#46 was chosen as the potential chromosome donor strain, since it carries the *HPH* hygromycin-resistance marker on chr^RC^. Spores from this strain were mixed with spores from three different ‘recipient’ strains: Fo47, Fol4287 and Fom001, all tagged by random insertion of the *BLE* zeocin-resistance gene. Double-resistant colonies were recovered only in the combination with Fo47. Nine such strains were saved and used for further analysis. All were shown by PCR to contain both *HPH* and *BLE* genes.

**Figure 5:**
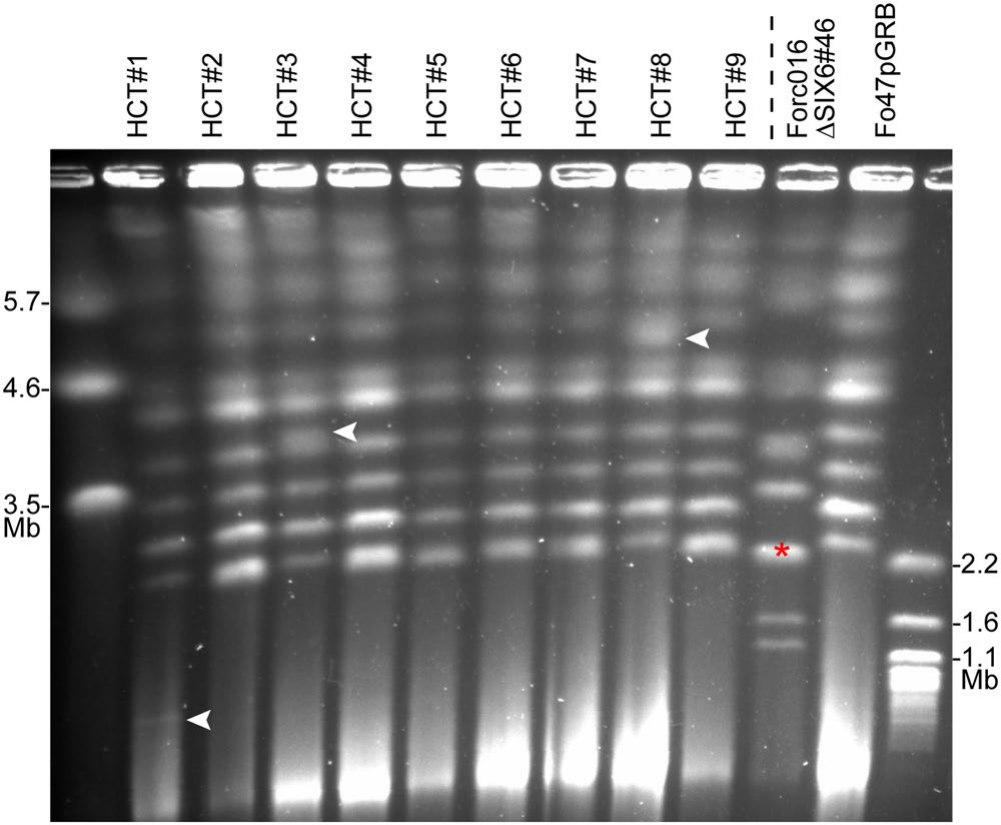
Nine strains derived from a HCT experiment between Forc016*∆SIX6*#46 and Fo47pGRB contain new chromosomes in the Fo47 background. Lanes 1–9 show the karyotype of HCT-derived strains, resembling that of Fo47pGRB (lane 11). Most of these strains have a double band at the size of chr^RC^ (~2.5 Mb), marked in Forc016*∆SIX6*#46 with a red asterisk (lane 10). Strains #1, #3 and #8 do not have this double band, but instead have at least one novel chromosome that is not found in either parental strain (white arrowheads). The left and right lanes show the karyotypes of *S*. *pombe* and *S*. *cerevisiae*, respectively, applied as markers. This image is cropped, the original gel photograph can be found in Supplementary Fig. S12.

To assess whether indeed chr^RC^ from Forc016 had been transferred to Fo47, a CHEF gel was run (Fig. 5). This revealed that all nine double-resistant strains displayed the karyotype of Fo47, with an additional chromosome presumably resulting from horizontal chromosome transfer (HCT). In the cases of HCT-derived strains #2, #4, #5, #6, #7 and #9 this chromosome is similar in size to a chromosome in the Forc016 donor strain (~2.5 Mb). However, since it is roughly the same size as the smallest chromosome of Fo47, the two co-migrated through the gel, resulting in a band with double intensity. In the three other cases (HCT #1, #3 and #8), this double band was absent and instead other new chromosomes were observed (Fig. 5, white arrowheads).

**Figure 6:**
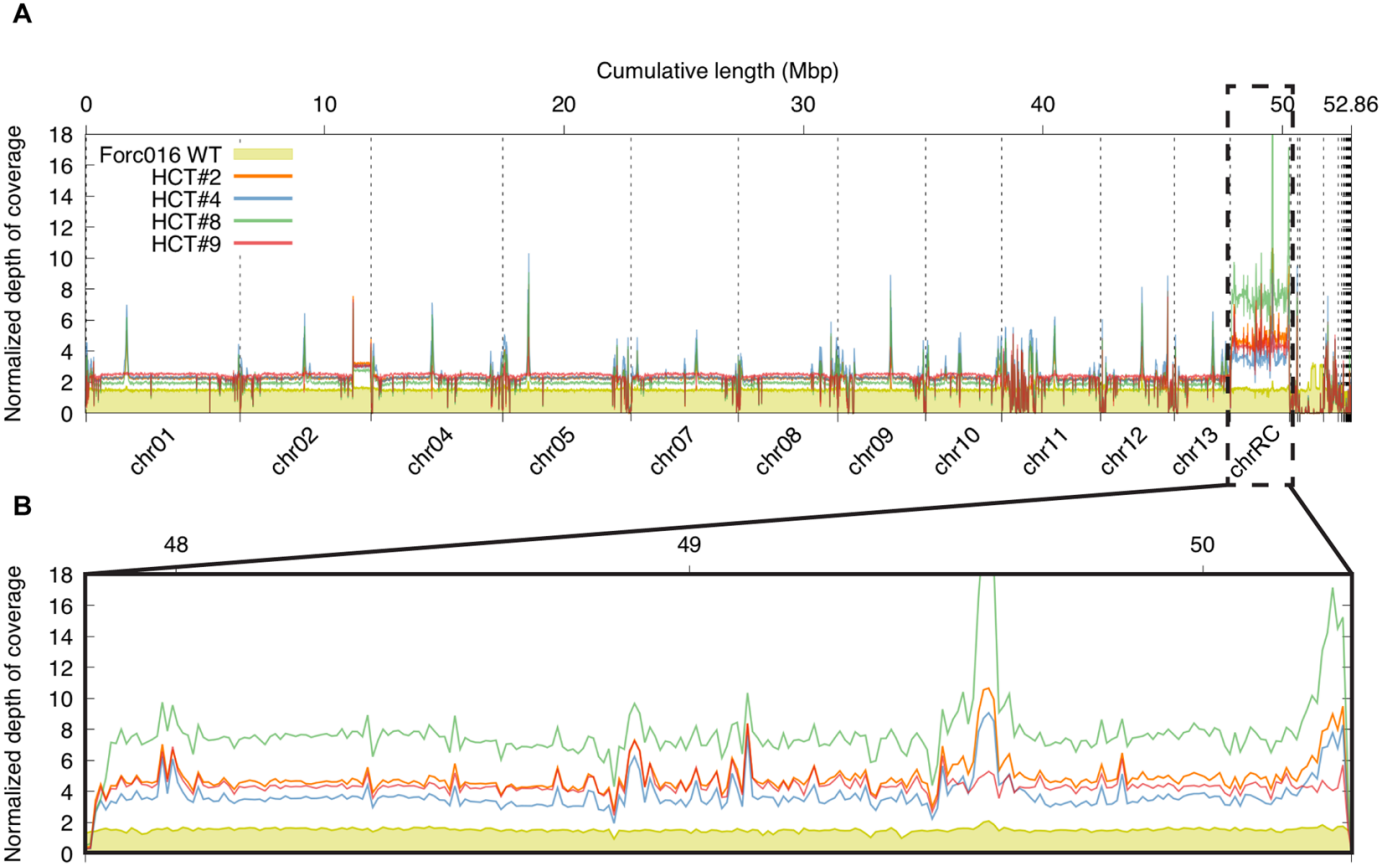
Normalized Illumina read mapping to the SMRT assembly of Forc016 confirms horizontal transfer of chr^RC^ in a Fo47 background. (**A**) Reads mapped more abundantly to the transferred chr^RC^ sequence than the rest of the assembly. (**B**) HCT strains #2, #4, #9 showed a relative (compared to total # mapped reads) depth of coverage of ~4 on chr^RC^, whereas the relative coverage of HCT#8 sequences was ~8 along the entire chromosome. This indicates a chromosomal duplication, in accordance with the ~5Mb-sized band in the CHEF gel in Fig. 5.

Stringent Illumina short-read mapping of HCT strains #2, #4, #8 and #9 on the Forc016 assembly showed that these strains indeed contain the full chr^RC^ sequence but no other Forc016-derived sequences (the core genomes of Forc016 and Fo47 have an average SNP density of 0.4% (more towards the telomeres), reducing mapping of reads to ~85%) (Fig. 6). This confirms that chr^RC^ now resided in a Fo47 core genome background. Remarkably, HCT#8 showed a relative depth of coverage of chr^RC^ about two times higher than the other HCT strains, indicating that the chr^RC^ sequences are present twice. Since a double band at the expected size of chr^RC^ (~2.5 Mb) is missing in this strain but a double-sized band of ~5 Mb is visible (Fig. 5), this duplication appears to have resulted in a single chromosome twice the size of chr^RC^. From the normalized read coverage of HCT strains #1 and #3 it is clear that large-scale rearrangements also took place along chr^RC^ in these strains (Fig. S7). Assembly of the mitochondrial genome with GRABb^17^ and comparison to the mitogenomes of Fo47 and Forc016 showed that the mitochondrial DNA of the Fo47 acceptor strain had been retained in all cases (data not shown).

### Chr^RC^ is capable of turning Fo47 into a cucurbit root rot pathogen

**Figure 7:**
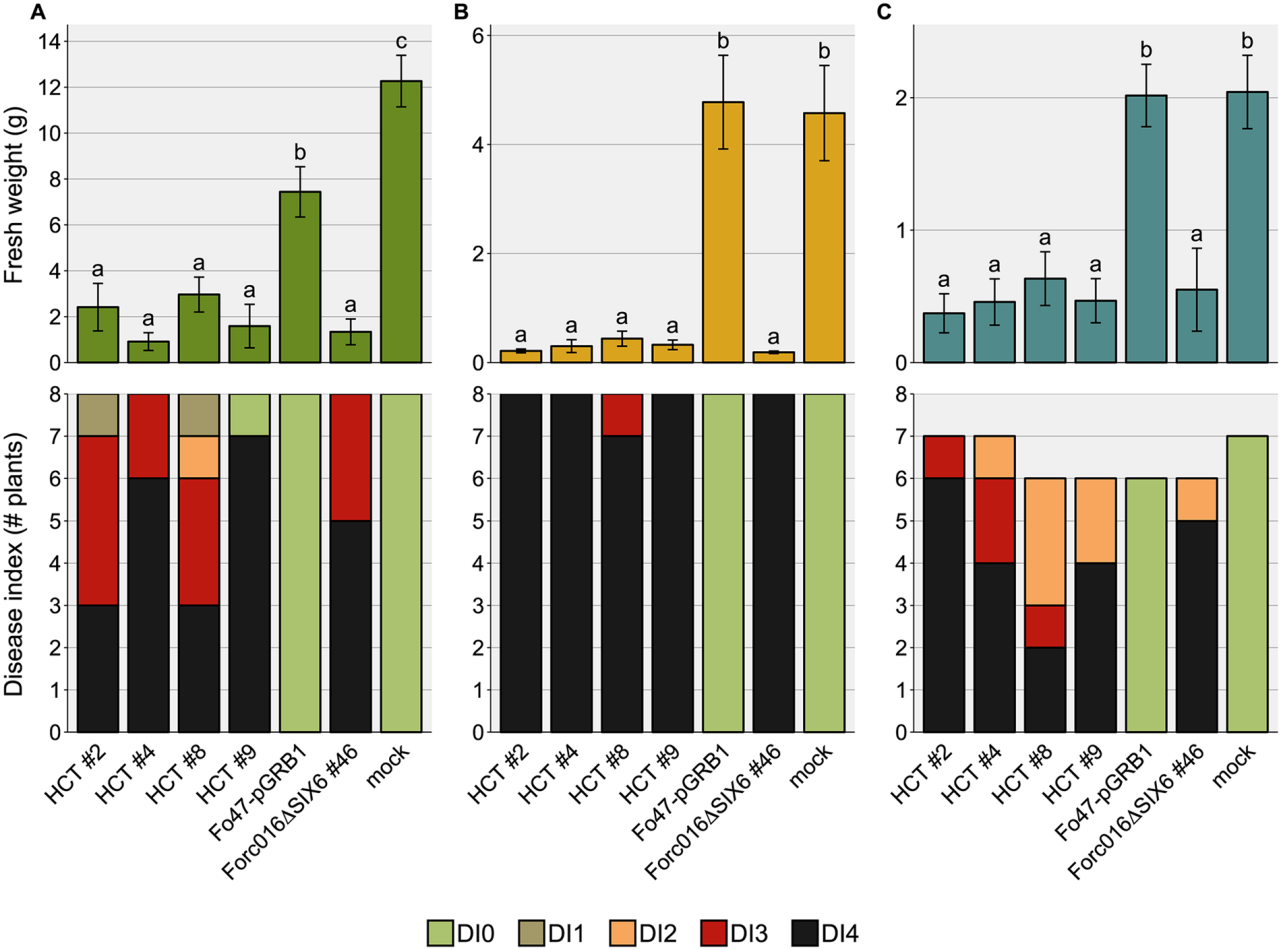
Horizontal transfer of chr^RC^ transforms the non-pathogenic strain Fo47 into a root and shoot rot pathogen of cucurbits. Fresh weight (±S.E.) and disease index (DI) of (**A**) cucumber, (**B**) melon and (**C**) watermelon plants were scored 14 days post inoculation. An ANOVA followed by a Tukey HSD test (p < 0.05) was performed to determine the significance of differences in the fresh weight measurements (significance categories shown with letters above the bars). Under the tested conditions (10^7^ sp/ml, 20 °C), HCT strains #2, #4, #8 and #9 caused a similar level of disease severity in all three cucurbit species as the chr^RC^ donor strain (Forc016*∆SIX6*#46).

To assess whether the HCT-strains, carrying chr^RC^ in a Fo47 background, are pathogenic on cucurbits, a bioassay was performed on cucumber, melon and watermelon plants. This time, the assay was done under conditions ideal for Forc infection, with a relatively low ambient temperature of 18–20 °C. All four tested strains (#2, #4, #8, #9) caused abundant symptom development in each of the three plant species, comparable to the control strain, Forc016*∆SIX6*#46 (Fig. 7; Fig. S8). This shows that the biocontrol strain Fo47 can be transformed into a *radicis*-*cucumerinum* strain, capable of infecting multiple host plants and causing root and shoot rot, by a single chromosome of Forc (Fig. S8).

### Chr^RC^ and the two smaller accessory chromosomes are conditionally dispensable

**Figure 8:**
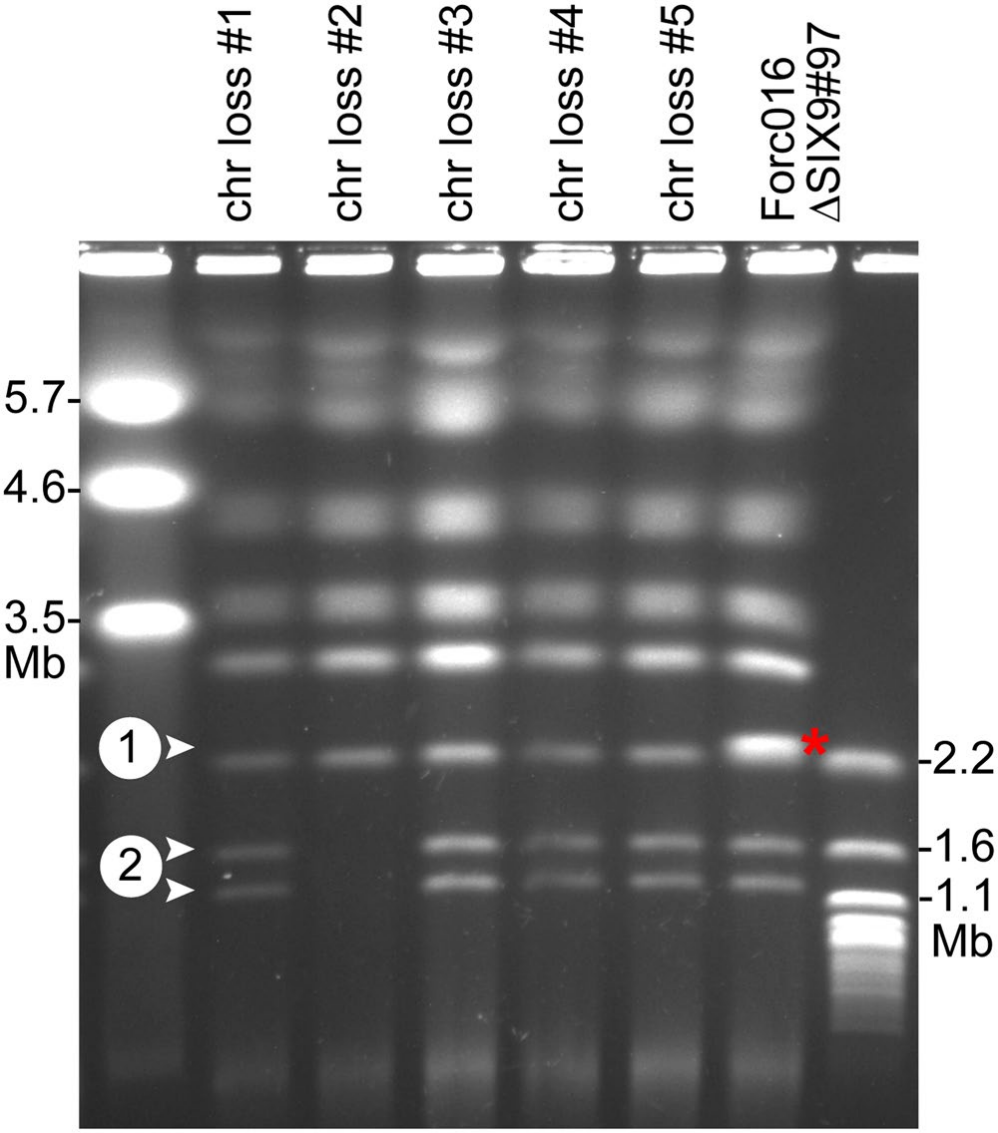
Chr^RC^ and the two smaller accessory chromosomes of Forc016 are conditionally dispensable. Electrophoretic karyotyping shows complete absence of chr^RC^ (marked with a red asterisk in lane 6) in all five hygromycin sensitive strains (arrowhead 1, lanes 1–5). Additionally, chromosome loss strain #2 displays absence of the two smallest chromosomes (arrowheads 2). The left and right lanes show the karyotypes of *S*. *pombe* and *S*. *cerevisiae*, respectively, applied as markers. This image is cropped, the original gel photograph can be found in Supplementary Fig. S13.

**Figure 9:**
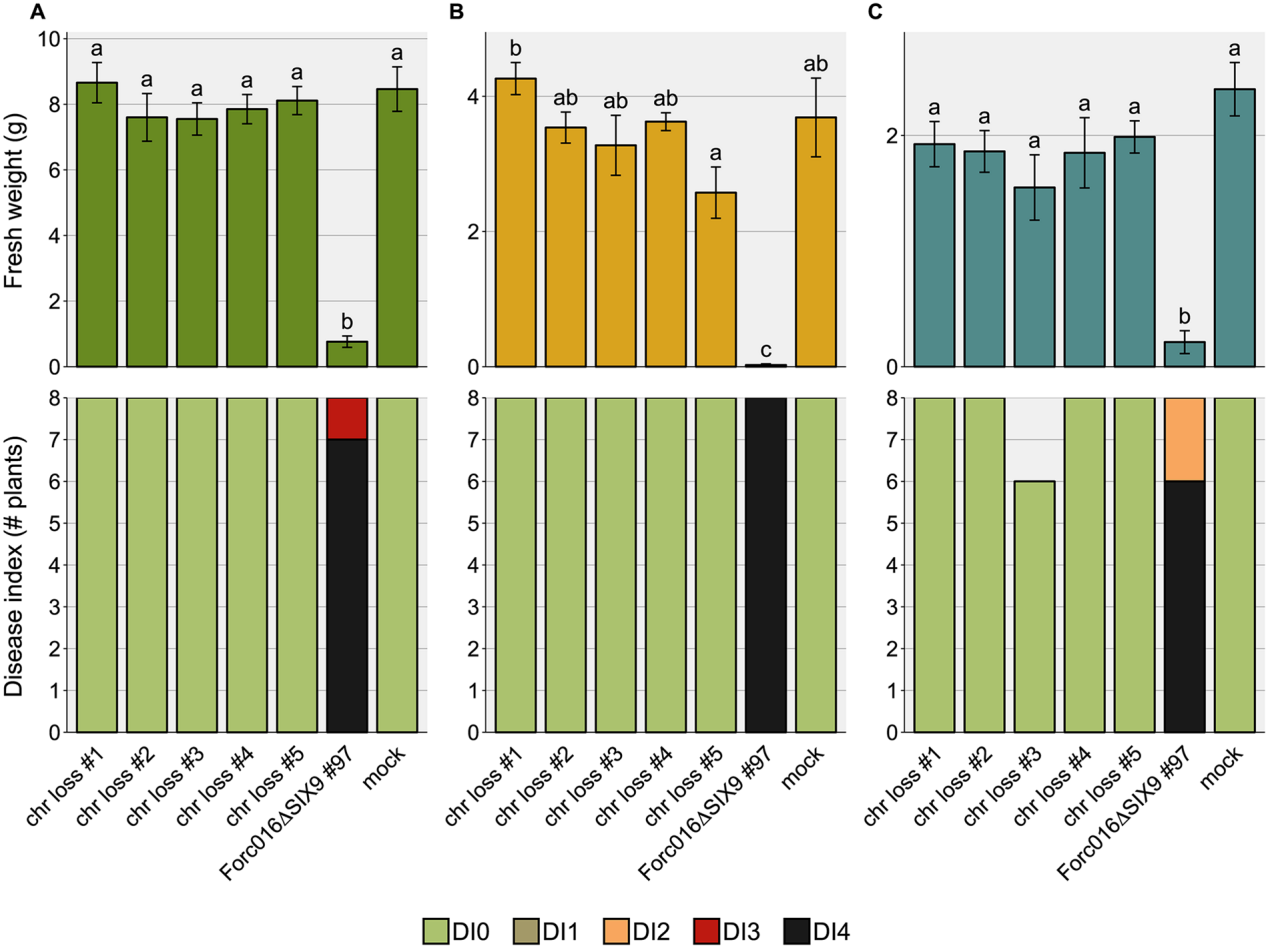
Forc016 strains without chr^RC^ are completely avirulent. Fresh weight (±S.E.) and disease index (DI) of (**A**) cucumber, (**B**) melon and (**C**) watermelon plants were scored 14 days post inoculation. An ANOVA followed by a Tukey HSD test (p < 0.05) was performed to determine the significance of differences in the fresh weight measurements (significance categories shown with letters above the bars). Under the tested conditions (10^7^ spores/ml, 20 °C), none of the chromosome loss strains were able to cause disease symptoms in cucurbit plants, while their parent strain (Forc016∆*SIX9*#97) was.

Incubation of the Forc016*∆SIX9* strain (harboring the *HPH* hygromycin resistance gene on chr^RC^) in medium containing 12.5 µg/ml benomyl yielded five strains that had become hygromycin sensitive, indicating a loss of the genomic region containing the *HPH* gene. Electrophoretic karyotyping showed that in all five cases, chr^RC^ had been completely lost (Fig. 8, Fig. S9). Chromosome loss strain #2 had additionally lost the two smaller accessory chromosomes of ~1.1 and 1.6 Mb. When tested in a bioassay, none of these strains caused symptoms in any of the three tested host plants (Fig. 9, Fig. S10). No growth rate alteration compared to their parent strain was found when the strains were grown on CDA or PDA medium. This demonstrates that chr^RC^ is required for pathogenicity of Forc016 towards multiple cucurbit species.

## Discussion

We show that Forc016 has 11 core chromosomes, one horizontally transferrable 2.446 Mb pathogenicity chromosome (chr^RC^) and two smaller accessory chromosomes. We conclude that chr^RC^ is necessary and sufficient for the root- and shoot-rot phenotype in several cucurbit species when infected by Forc. More specifically, the middle region of chr^RC^, which differs from Fom001’s homologous chromosome, might play a decisive role in both the extended host range and rotting symptoms caused by Forc.

### Are effectors important for Forc pathogenicity?

Since the identification of *SIX1*, the first effector gene from *F*. *oxysporum* f.sp. *lycopersici* ^26^, thirteen more *SIX* genes have been described^10, 27^. For several of these, including *SIX6*, a role in virulence has been shown^12, 26, 28–31^. *SIX6* homologs have been found in Fo f.sp. *lycopersici*, *cucumerinum*, *radicis*-*cucumerinum*, *melonis*, *niveum*, *pisi*, *passiflorae*, *cubense* and *vasinfectum* ^9, 32–34^, as well as in Fo f.sp. *momordicae*, *luffae*, *Fusarium hostae* ^35^ and in *Colletotrichum* spp.^36^. Strains belonging to the same *forma specialis* typically have the same sequence types for effector genes, even when core genes are not identical in sequence^9^. This is likely the result of horizontal inheritance of dispensable genomic regions^34^ and is corroborated by the incongruent phylogeny of *SIX* genes compared to the housekeeping gene *EF1α* reported by Rocha *et al*.^37^. Interestingly, Forc shares its *SIX6* sequence type with strains belonging to Fo f.sp. *melonis* (Fom) as well as some – but not all – Fo f. sp. *niveum* (Fon) strains^9^. This may be indicative of a (partially) shared ancestry of cucurbit-infection between these *formae speciales*.

Deletion of *SIX6* in Fol marginally compromises virulence in the Fol-tomato pathosystem. Additionally, Six6 suppresses I-2-mediated cell death upon transient expression in *N*. *benthamiana*, but does not compromise the activity of other cell-death-inducing genes^12^. Three individual Fol strains with a partial deletion of chromosome 14, thereby losing *SIX6*, *SIX9* and *SIX11*, as well as *ORX1* encoding an in xylem-secreted oxidoreductase, did not show a significant reduction in disease severity, indicating that these genes are largely dispensable for Fol pathogenicity^14^. In the Fon-watermelon pathosystem, however, Six6 has been reported to be involved in virulence^38^.

We find a clear reduction in virulence of three independent Forc strains in which the *SIX6* locus was disrupted (Fig. 4). However, this phenotype could only be observed at relatively high ambient temperatures (25 °C) in cucumber, while at lower temperatures all plants died. In contrast to most wilt-causing Fo pathogens like Fol, Foc, Fom and Fon, Forc symptoms develop most efficiently at temperatures below 20 °C^6, 39, 40^, particularly during seedling infection, when plants may be under physiological stress^6^. We conclude that Six6 contributes to virulence only under non-optimal conditions and only in cucumber.

The other tested effector candidate knockout strains (Δ*SIX9* and Δ*SMP1*) did not display a reduction in virulence towards cucumber, melon or watermelon compared to Forc-wt and ectopic transformant strains. Six9 and Smp1 are therefore, by themselves, not important for disease development caused by Forc.

### HCT of chr^RC^ contributes to genome evolution in Fo

HCT has so far been described for Fol chromosomes 7, 8, 14 and the smallest chromosome of Fol007^15, 16^, but was until now not shown for other *formae speciales* of Fo. Non-pathogenic recipient strain Fo47 became pathogenic towards tomato upon receiving Fol chromosome 14, albeit less than the Fol donor strain. A higher aggressiveness of HCT-strains was observed when another accessory chromosome co-migrated, potentially due to the influence of transcription factors located on that chromosome^19^. Interestingly, two copies of *FTF1*, a transcription factor associated with effector gene expression^19, 20, 41^, are located in the effector-rich central part of chr^RC^ (Fig. 3), potentially indicating a partial transcriptional autonomy of chr^RC^.

Horizontal transfer of chr^RC^ was accomplished with Forc016∆*SIX6* as a donor and Fo47 as a recipient strain. Nine double-drug resistant colonies were recovered after co-cultivation and electrophoretic karyotyping of these HCT strains (Fig. 5) showed that six strains gained chr^RC^ while three strains (#1, #3, #8) had undergone chromosome rearrangements. HCT strain #8 had a double relative coverage of chr^RC^ compared to that of the other strains and a band at twice the size of chr^RC^ (~5 Mb, Fig. 5) in its electrophoretic karyotype. The chromosome apparently duplicated but remained present as a single entity, pointing to a high level of genome plasticity.

A recent study by Vlaardingerbroek *et al*.^16^ also showed chromosomal plasticity in horizontal transfer experiments of the Fol pathogenicity chromosome. Transformation for marker insertion on this chromosome resulted in a larger (estimated 250 kb) pathogenicity chromosome in a donor strain that was used for HCT towards Fo47. Selection for loss of this chromosome in another study^14^ resulted in several strains that only partially lost the chromosome. Interestingly, deletions within a chromosome and chromosomal breaks appeared to happen non-randomly at so-called ‘deletion hotspots’. Whether something similar happened to HCT strains #1 and #3 in this study remains to be seen. It is clear that genomes of *F*. *oxysporum*, particularly the accessory parts defining host virulence, are highly plastic. This could result in accelerated genetic diversification, possibly facilitating adaptation to new environments including new host plants.

In Forc, two vegetative compatibility groups (VCGs) have been described: VCG0260 (to which Forc016 belongs) and VCG0261^42^. RAPD fingerprinting analyses and concatenated sequence alignment of 1195 conserved core genes showed that the two VCGs are very similar and appear to be clonally related^9, 39^. The other two sequenced Forc strains, Forc031 (VCG0261) and Forc024 (VCG0260), both possess the chr^RC^ sequence and their effector gene content is nearly identical^9^. Interestingly, large parts of chr^RC^ were also identified in two out of three sequenced Fom VCGs, including Fom001 (VCG0136, Fig. 3), whose core genome is highly similar to that of the Fol4287 reference strain and other Fol strains in VCG0030^9^. This is a strong indication that the Forc and Fom pathogenicity chromosomes evolved from a shared ancestor. Integration of the highly diverse central region in an ancestral chromosome from an unknown source potentially gave rise to chr^RC^ and the new *forma specialis radicis*-*cucumerinum*. The suite of candidate effector genes found in Forc, concentrated in the central region of chr^RC^ (Fig. 3), is most similar to that of strains belonging to Fo f.sp. *cucumerinum* ^9^. Systematic comparative and functional analysis of the accessory genomic regions of multiple cucurbit-infecting *formae speciales* will be necessary to reconstruct the evolutionary paths that led to host-specificity of Fo towards this plant family.

The wider host range of Forc compared to Fom could be caused by the absence of avirulence genes. *SIX1* has been reported as an avirulence gene in the Fol-tomato interaction (*AVR3*) and could potentially be recognized by cucumber and watermelon, triggering a defence response by these plants upon colonization by Fom. This is the only *SIX* gene homolog that is consistently present in the Fom genome but is not found in Forc.

### Assembly of highly repetitive genomes benefits from long-read sequencing technology

Repetitive regions, including centromeres of *Fusarium*, are difficult to assemble using short-read sequencing technologies such as Illumina. *F*. *oxysporum*’s compartmentalized genome is a good example of a genome that can benefit greatly from longer read sequencing techniques, such as the PacBio SMRT sequencing technology employed here (median read length 15 kb) as well as the development of novel technologies such as Oxford Nanopore sequencing^43^. Manual curation of the assembly improved it to the point where five chromosomes are complete (telomere-to-telomere), fifteen contigs have telomeric repeats on only one end and 20 contigs are left with no telomeric repeats on either end. The estimated chromosome count of Forc016 is 14, which is most clearly visible in Fig. 6 where the separation in the 1–1.5 Mb region shows that Forc016 possesses two small accessory chromosomes. Considering their size and accessory-like appearance (high TE and low gene content), these possibly correspond to contig 53 and the non-conserved region that is probably erroneously attached to chromosome 11 in the assembly. This is supported by the read mapping of chromosome loss strain #2, where no coverage was found for the mentioned two regions, as well as contig 3 and several of the smaller unplaced contigs (Fig. S9B). This strain lost these two chromosomes in addition to chr^RC^, showing that they are conditionally dispensable. Moreover, the pathogenicity of the chr^RC^ chromosome transfer strains demonstrates that the two smallest Forc016 chromosomes are not required for pathogenicity. Comparison of the read coverage from chromosome loss strain #2 to wildtype will allow us to see which contigs belong to these chromosomes.

Even though PacBio SMRT sequencing is a great improvement to short-read technologies, it does not as yet allow for completely closed assemblies for *F*. *oxysporum* if not combined with other techniques like optical mapping^44, 45^. Nevertheless, the assembly of the core chromosomes as well as the pathogenicity chromosome of Forc016 were of sufficient quality to answer the biological questions addressed here.

### Conclusions

We report here the near-complete genome assembly of Fo f.sp. *radicis*-*cucumerinum* strain Forc016 and horizontal transfer of its pathogenicity chromosome, chr^RC^, to the non-pathogenic strain Fo47. This is the first time HCT has been accomplished using a donor strain from a *forma specialis* other than *lycopersici*. The virulence of the progeny strains deriving from this experiment is identical to that of the Forc chromosome donor, indicating that chr^RC^ is sufficient for root and shoot rot disease development. Complete loss of virulence of the five strains that lost chr^RC^ shows that chr^RC^ is also required for pathogenicity of Forc016. The experimental evidence presented here provides compelling confirmation that horizontal transfer of genetic material plays a crucial role in the adaptation to new host ranges of pathogenic isolates within the *F*. *oxysporum* species complex.

## Methods

### Fungal strains

*F*. *oxysporum* strains Forc016 (‘33’; CBS141123)^9, 46^ and Fom001 (NRRL26406)^47^ were sequenced with SMRT sequencing technology. Fo47pGRB1^16^ was used as a chromosome recipient in HCT experiments.

### Cloning

pPDh was constructed by introducing a *KpnI*-*KpnI* fragment containing a multiple cloning site (MCS) and the *eGFP* coding sequence followed by the *SIX1* terminator sequence, amplified from pPZP200-pSIX1GFP^48^, into the binary vector pRW2h^49^. Additionally, a *HindIII*-*HindIII* fragment containing a MCS and the Herpes Simplex Virus thymidine kinase (*HSVtk*) gene under the control of the *C*. *heterostrophus* glyceraldehyde-3-phosphate dehydrogenase (ChGPD) gene promoter and the *N*. *crassa* β-tubulin gene terminator was inserted into the vector as a conditional negative selection marker against ectopic transformants^50^.

For knockout constructs, two ~1 kb fragments flanking the gene of interest were amplified using the primers listed in Table S2. The fragments were digested with *PacI*-*SpeI* and *AscI*-*Sbf1* (*SIX6*; *SMP1*) or *PacI*-*SpeI* and *AscI*-*BstEII* (*SIX9*) and subsequently inserted on either side of the *GFP / HPH* cassette of pPDh.

### Forc gene knockout

*F*. *oxysporum* strain Forc016 was transformed by *Agrobacterium* mediated transformation as described previously^51^. Following monosporing of hygromycin-resistant colonies, the transformants were grown in 96-well plates containing in each well 150 µl PDB supplemented with hygromycin and 5 µM 5-Fluoro-2-deoxyuridine (Alfa-Aesar) for pre-selection of *in locus* transformation^50^. Successful knockout of the genes was confirmed by PCR, using primers inside the T-DNA and outside the 1 kb flanking region.

### Disease assays

Pathogenicity tests were performed using the root dip method^52^. In short, conidia were isolated from five-day-old cultures NO_3_-medium (0.17% yeast nitrogen base, 3% sucrose, 100 mM KNO_3_) by filtering through miracloth (Merck; pore size of 22–25 μm). Spores were centrifuged, resuspended in sterile MilliQ water, counted and brought to a final concentration of 10^6^ (effector KO assay) or 10^7^ spores/mL (chromosome transfer and loss assays). When the first true leaves were emerging (after ±10 days), 6–8 seedlings per treatment were uprooted, inoculated, individually potted and kept at 25 °C (effector KO assay) or 20 °C (HCT assay) in the greenhouse. The following plant cultivars were used: *Cucumis sativus* cv. Paraiso, *Cucumis melo* cv. Cha-T, *Citrullus lanatus* cv. Black Diamond. Two weeks after inoculation, disease was scored using a disease index from 0–4 (0, no symptoms; 1, slight root rot symptoms, only at tip of main root; 2, root rot symptoms and stem lesions visible aboveground; 3, very clear root rot symptoms of the entire root system, often with a large lesion extending above the cotyledons; 4, plant either dead or very small and wilted).

### Chromosome transfer and loss

Chromosome transfer from Forc016*ΔSIX6*#46 to Fo47pGRB^16^ was performed through co-cultivation of the strains^53^. 1 × 10^5^ microconidia from each of the two strains were mixed and co-incubated on PDA plates for six days. Newly formed spores were washed from the co-incubation plate using 5 ml sterile MilliQ, filtered through sterile miracloth and pipetted on a double selective PDA plate containing 0.1 M Tris pH 8 supplemented with 100 µg/ml hygromycin (Duchefa) and 100 µg/ml zeocin (InvivoGen). Double drug resistant colonies were selected after six days and monospored by spreading on a fresh plate supplemented with both antibiotics. After two days of growth, single-spore colonies were selected and transferred to fresh plates.

Chromosome loss was induced as previously described^54^ with some modifications. Forc016*∆SIX9*#97 was grown on PDA supplemented with hygromycin for 4 to 10 days. A Forc016 *∆SIX9*#97 mycelial agar block was incubated in M100 broth^54^ containing 12.5 µg/ml benomyl (methyl 1-(butylcarbamoyl)-2-benzimidazolecarbamate, Aldrich) for 4 days, 175 rpm at 25 °C. The culture was filtered through sterile miracloth. Conidia were collected by centrifugation and resuspended in 5 ml sterile water. 100 µl of a 100-fold dilution of conidia suspension was spread on M100 plates containing 0.04% Triton X-100 (Sigma). The plates were overlaid with a sterile filter paper and plates and conidia were incubated at 25 °C for 2 days. The paper was transferred from M100 plates to PDA with hygromycin. After 1–2 days, the paper was removed and the colonies surviving only on M100 were selected and transferred to fresh PDA plates for further analysis.

### Electrophoretic karyotyping and Southern analysis

Preparation of protoplasts and running of pulsed-field gel elecrophoresis was performed as described previously^16, 55^. *F*. *oxysporum* was cultured in 100 ml NO3 medium for five days. Next, microconidia were collected by filtration through a double layer of sterile miracloth. 5 × 10^8^ spores were transferred to 40 ml PDB (BD Biosciences) and grown for 13 h at 25 °C, followed by incubation at 30 °C for 13–16 h in MgSO_4_ solution (1.2 M MgSO_4_, 50 mM sodium citrate, pH 5.8) supplemented with 50 mg/ml Glucanex (Sigma). Protoplasts were filtered through a double layer of miracloth, collected by centrifugation and cast in InCert agarose (Lonza) plugs at a concentration of 1 × 10^8^ protoplasts per ml. Plugs were treated with Pronase E and chromosomes were separated by running for 260 hours in 1% Seakem Gold agarose (Lonza) at 1.5 V/cm in a CHEF-DRII system (Biorad) in 0.5 × TBE at 4 °C with switch times between 1200 and 4800 s. The gels were stained with ethidium bromide and de-stained using 0.5 × TBE.

DNA was blotted to a Hybond N + membrane (Amersham Pharmacia) by alkaline transfer with 0.4 N NaOH. A 793 bp PCR product containing the Fol*SIX6* open reading frame was generated with primers FP1490 and FP1491 (Table S2). This fragment was radioactively labelled with [α^32^P]-dATP using the DecaLabel DNA labeling kit (Thermo Scientific). Hybridization was performed overnight at 65 °C in Church and Gilbert buffer containing 0.5 M phosphate, 7% SDS and 1 mM EDTA at pH 7.2. Blots were washed at 65 °C with 0.5 × SSC, 0.1% SDS. The position of chromosomal sequences to which the *SIX6* probe hybridized was visualized by phosphoimaging (Storm 840, Molecular Dynamics).

### DNA isolation, genome sequencing and assembly

DNA isolation was performed on freeze-dried mycelium ground in liquid nitrogen as starting material, using multiple rounds of phenol-chloroform extraction and precipitation, as well as treatment with RNase A and proteinase K.

SMRT sequencing was performed at Keygene N.V. (Wageningen, the Netherlands). PacBio libraries were prepared and size-selected at ~20 Kb using Blue Pippin prep. Sequencing of 5 SMRT cells was performed using the P6-C4 polymerase-chemistry combination, ≥4 hr movie time, stage start. This resulted in a sum of 4772 Mb (Forc016) and 4846 Mb (Fom001) filtered data. *De novo* assembly was performed with the Hierarchical Genome Assembly Process v3 (HGAP.3, Pacific Biosciences) within the SMRT Portal environment (v1.87.139483). Default values were kept and the expected genome size was set to 60 Mb.

The raw assembly was manually improved by removing contigs originating from mtDNA and rDNA repeats. Two contigs that ended in telomeric repeats on one end and rDNA repeats on the other were joined together with in total 97 rDNA repeats in between (based on Illumina read mapping and coverage estimation on 10 rDNA repeats). Chromosome 13 could be reconstructed by joining two contigs that showed conserved synteny in Fom001 and the SMRT assembly of *F*. *subglutinans*. The two contigs were merged at the position of an overlapping region of 13,396 nt.

The mitochrondrial DNA was assembled from Illumina reads using GRAbB^17^ by specifying the mitochondrial genome of *F*. *oxysporum* F11 as reference and employing SPAdes as assembler. Annotation of the mitogenome was performed as described in Brankovics *et al*. (submitted) using a combination of MFannot (http://megasun.bch.umontreal.ca/cgi-bin/mfannot/mfannotInterface.pl), tRNAscan-SE^56^, NCBI ORFfinder (https://www.ncbi.nlm.nih.gov/orffinder), InterPro^57^ and CD-Search^58^.

Illumina sequencing (150 bp paired-end, insert size ~450 bp) of HCT strains was performed on a HiSeq. 2500 machine by the Hartwig Medical Foundation (Amsterdam, the Netherlands) at ~100X coverage, resulting in 5.0–5.6 Mb of sequence data per sample.

### Genome annotation

Repeats were identified with RepeatMasker v4.0.6 (with -engine ncbi -species “ascomycota”)^59^. Gene prediction was executed on the repeat-masked genome assembly by running BRAKER1 v1.9^60^, using RNA-seq read mappings (both *in vitro* and 10 days post inoculation *in planta* conditions) as additional evidence and supplying the following flags: –fungus –useexisting=“fusarium_graminearum”. Repeats and genes were counted over 50 kb windows along the genome.

InterProScan v5.18–57.0 was used to assign functional annotation (including GO terms) to predicted genes. In order to find overrepresented GO terms on chr^RC^ versus the whole genome, a hypergeometric test was performed on the GO term frequencies using the ‘phyper’ function in R. The p values were adjusted for multiple comparisons using ‘p.adjust’ and selecting the Bonferroni method in R. The results were visualized using REVIGO^61^.

### Read mapping and genome analysis

For coverage plots, reads were trimmed to remove low-quality bases and adapter sequences using fastq-mcf v1.04.807 (-q 20) and mapped against the Forc genome assembly with Bowtie2 v2.2.5 (DNAseq) or Tophat2 v2.1.0 (RNAseq). Optical duplicates were removed using PicardTools MarkDuplicates v2.7.1 and coverage per 10 kb (HCT plots) or 50 kb (circos plots) windows was calculated with the samtools v1.3.1 mpileup command.

Whole genome or chromosome alignments were performed using nucmer (with –maxmatch) from the MUMmer v3.23 package^62^. Comparison to the Fol4287 reference genome was done against an approximate chromosome-level assembly in which we concatenated scaffolds as assigned to chromosomes in refs 10 and 15, separated by 1000 Ns. We kept accessory regions of chromosomes 1 and 2 as separate sequences (for visualisation in Fig. 2E).

Identification of candidate effectors was done with BLASTN using the list of 104 candidates from van Dam *et al*.^9^ as a query fasta. Mimps were identified by searching the genome for a consensus sequence of the mimp inverted repeat (IR), “TT[TA]TTGCNNCCCACTGNN”. If two were found within 400 nt from each other in the correct orientation, they were marked as the ends of an intact mimp element.

### Data availability

The Whole-Genome Shotgun projects for the resequenced strains have been deposited at Genbank under the BioProjects PRJNA389503 and PRJNA389439. The genome assemblies can be found on GenBank under accession numbers MABQ01000000 (Forc016 Illumina assembly), MABQ02000000 (Forc016 SMRT assembly) and NJCY01000000 (Fom001 SMRT assembly). Raw SMRT sequence data, Illumina read data of the HCT and chromosome loss strains and RNAseq reads have been deposited into the Sequence Read Archive under the accession number SRP108975. Illumina paired-end read data for Forc016 is available under accession number SRP067515 (DNAseq).

## Electronic supplementary material

Supplementary Information

Video S1
