# Supplementary material for: A mobile pathogenicity chromosome in Fusarium oxysporum for infection of multiple cucurbit species
Source: Sci Rep. 2017 Aug 22;7:9042. doi: 10.1038/s41598-017-07995-y (PMC5567276; doi:10.1038/s41598-017-07995-y)
Supplement: Supplementary file 1 — Supplementary Information [file 41598_2017_7995_MOESM1_ESM.pdf]

Supplementary data to

A mobile pathogenicity chromosome in *Fusarium oxysporum* for infection of multiple cucurbit species

Peter van Dam[1], Like Fokkens[1], Yu Ayukawa[2], Michelle van der Gragt[1], Anneliek ter Horst[1], Balázs Brankovics[3], Petra M. Houterman[1], Tsutomu Arie[2], Martijn Rep[1]*

1. Molecular Plant Pathology, Swammerdam Institute for Life Sciences, University of Amsterdam, The Netherlands
2. Laboratory of Plant Pathology, Graduate School of Agriculture, Tokyo University of Agriculture and Technology (TUAT), Fuchu, Tokyo, Japan
3. Westerdijk Fungal Biodiversity Institute, Utrecht, The Netherlands

* Corresponding author
E-mail: m.rep@uva.nl

Supplementary Data

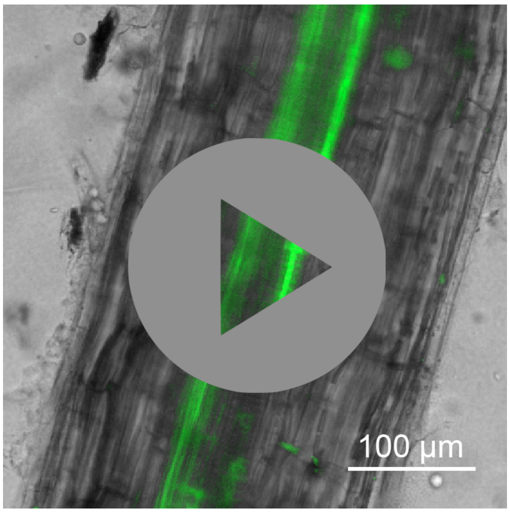

[Video as separate file online]

Video S1: Microscopic timelapse movie of GFP-tagged Forc016 shows the fungus colonizing a cucumber plant through the xylem tissue.
Nine day old cucumber seedlings were inoculated with a Forc016 strain that was transformed with the pPK2*hphgfp* construct (*Hyg^R-GFP* phusion protein under the control of the constitutive *gpdA* promoter) [62]. At 9 days post inoculation, the timelapse was recorded over an 8h20m time period with 1 minute-intervals, showing that Forc colonizes the plant like wilt-inducing strains of *F. oxysporum* do: by growing through the xylem vessels of the plant.

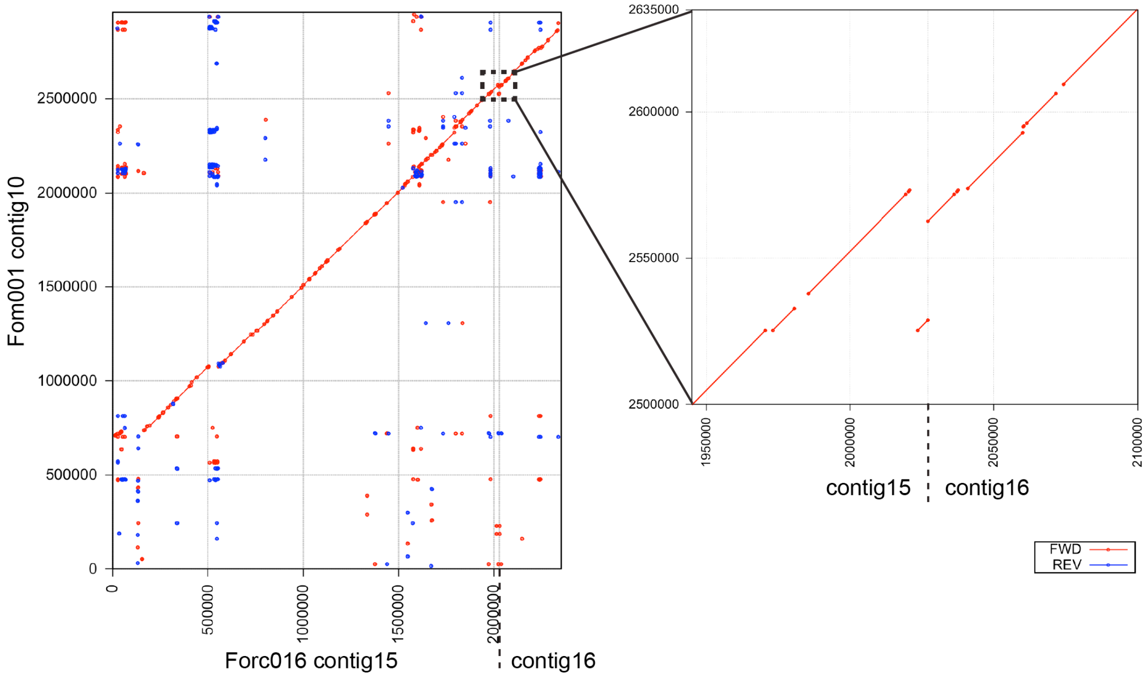

Fig S1: Two contigs in the Forc016 assembly, 15 and 16, display an overlap of 13,396 nt and are syntenic in Fom001, indicating that it is highly likely that they together form chromosome 13 in Forc016.

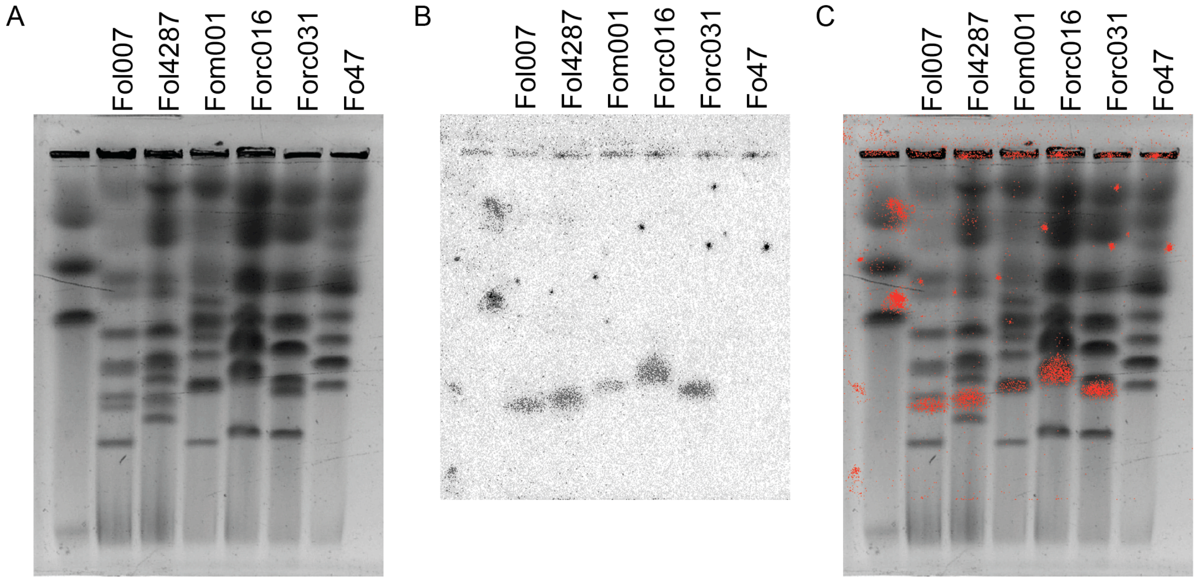

Fig S2: Radioactively labeled *SIX6* probe hybridizes at the location of *SIX6* in the CHEF gel run for Fol007, Fol4287, Fom001, Forc016, Forc031 but not Fo47, since it does not possess this gene.
(A) CHEF gel separation of the chromosomes of these strains. The left lane shows the marker *(Saccharomyces cerevisiae* chromosomes) with bands indicating 5.7, 4.6 and 3.5 Mb. **(B)** Southern hybridization signal using a *SIX6* probe. **(C)** Overlay of the Southern hybridization signal (in red) over the CHEF gel in figure A.

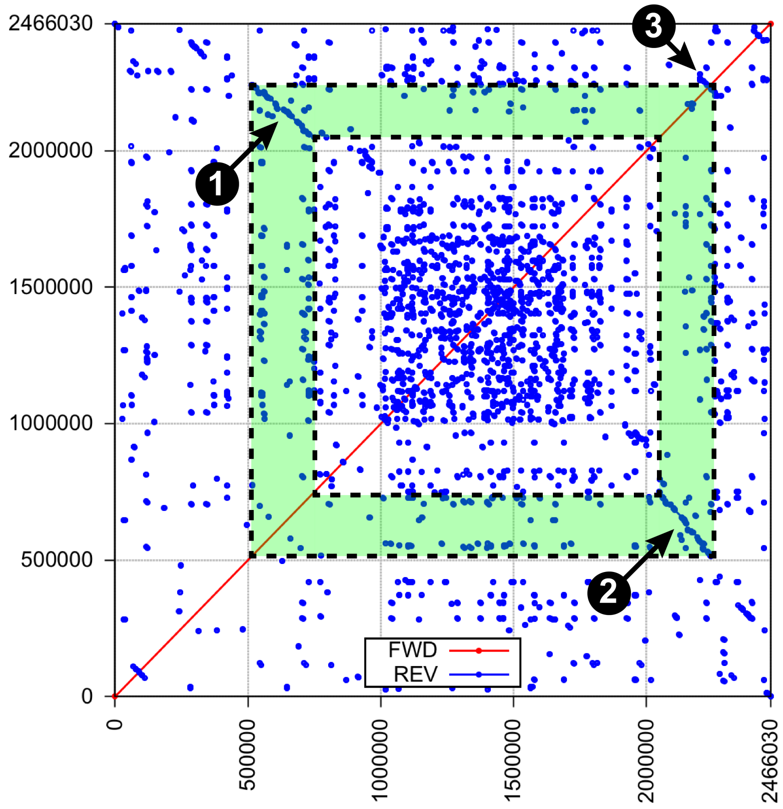

Fig S3: Nucmer comparison of Forc016's chr^{RC} to itself reveals that large repetitive regions are present around the middle region of the chromosome.
These repetitive regions are located on 500-700kb (arrow 1) and 2000-2200kb (arrow 2) of the chromosome, which resulted in a misassembled inversion of this sequence in the original assembly. This region (marked as a green box) was manually inverted in contig 13 and merged with contig 17 at the position of arrow 3.

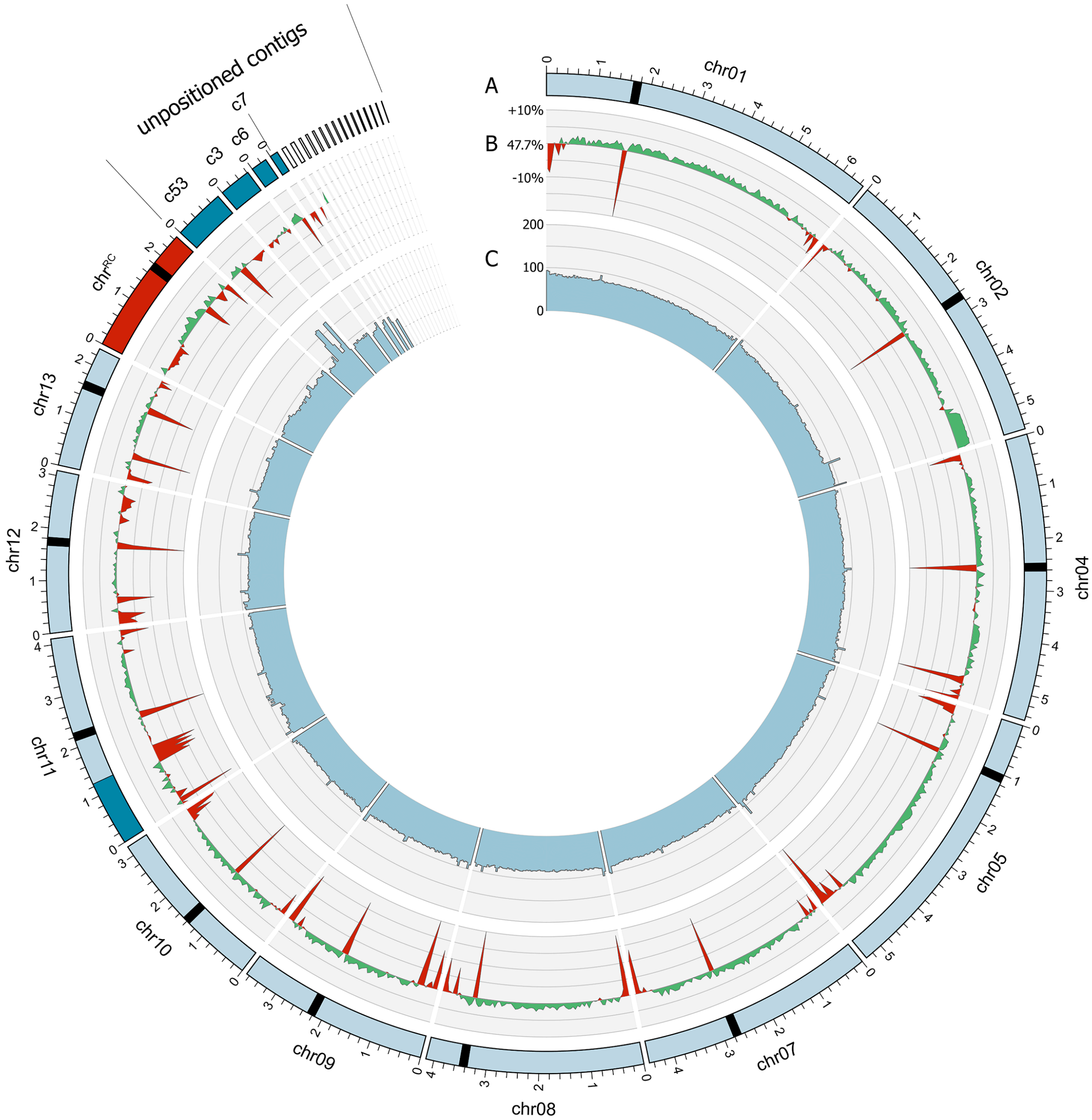

Fig S4: Visualization of the Forc016 genome assembly reveals centromeres in the assembly and two segmental duplications on contig 53.
The panels in this figure indicate **(A)** the karyotype of the assembly, with core chromosomes (light blue), accessory regions (dark blue) and the pathogenicity chromosome, chr^{RC} (red). Probable centromeres (characterized by low GC content, shown in **(B)**) are indicated with black blocks. **(C)** Read density levels calculated in 50kb windows for Illumina paired-end read mapping shows that almost the complete genome is covered at about 90X, with the notable exception being contig 53 which shows two large segmental duplications, roughly 220kb and 140kb in size.

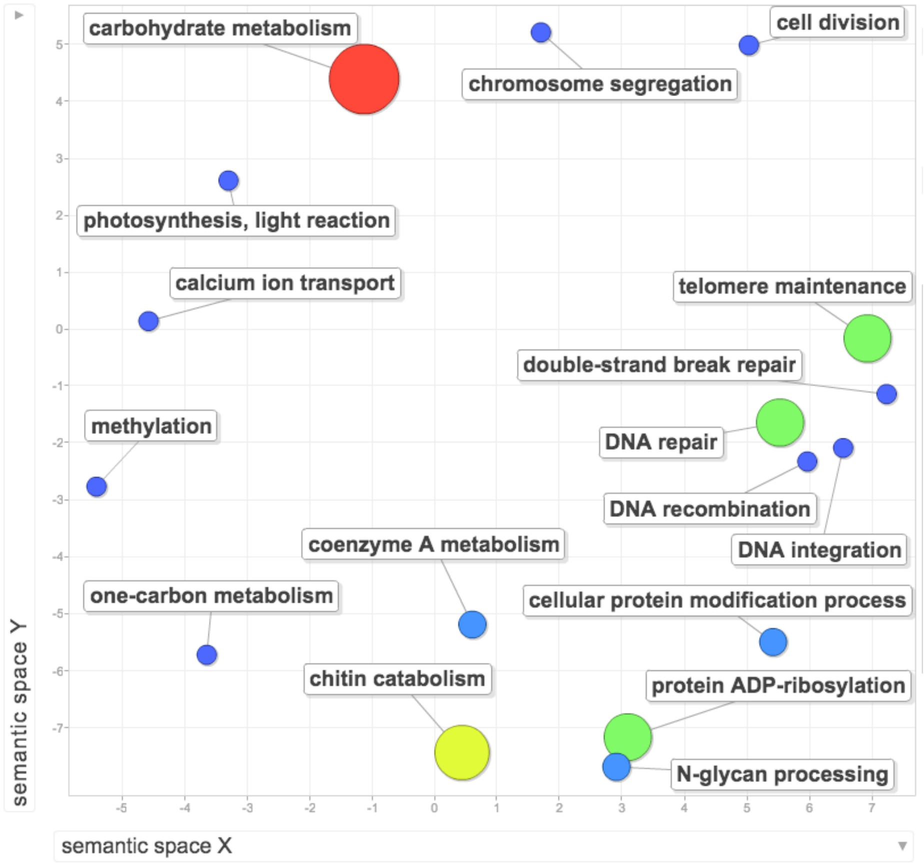

Fig S5: Overrepresented gene ontology (GO) terms on chr[RC] include genes related to carbohydrate metabolism, chitin metabolism, protein ADP-ribosylation and several groups related to DNA integrity (DNA repair, telomere maintenance, DNA recombination, DNA integration, chromosome segregation and cell division).

A hypergeometric GO term enrichment analysis ($p<0.05$) was performed to identify which types of genes (other than effectors) are overrepresented on chr[RC] compared to the rest of the Forc016 genome. Overrepresented GO terms related to Biological Process (P) were visualized using REVIGO (http://revigo.irb.hr/). The axes have no intrinsic meaning - REVIGO uses multi-dimensional scaling to reduce the dimensionality of a matrix of the GO terms' pairwise semantic similarities. Semantically similar GO terms should remain close together in the plot. See Table S2 for further details on the overrepresented genes.

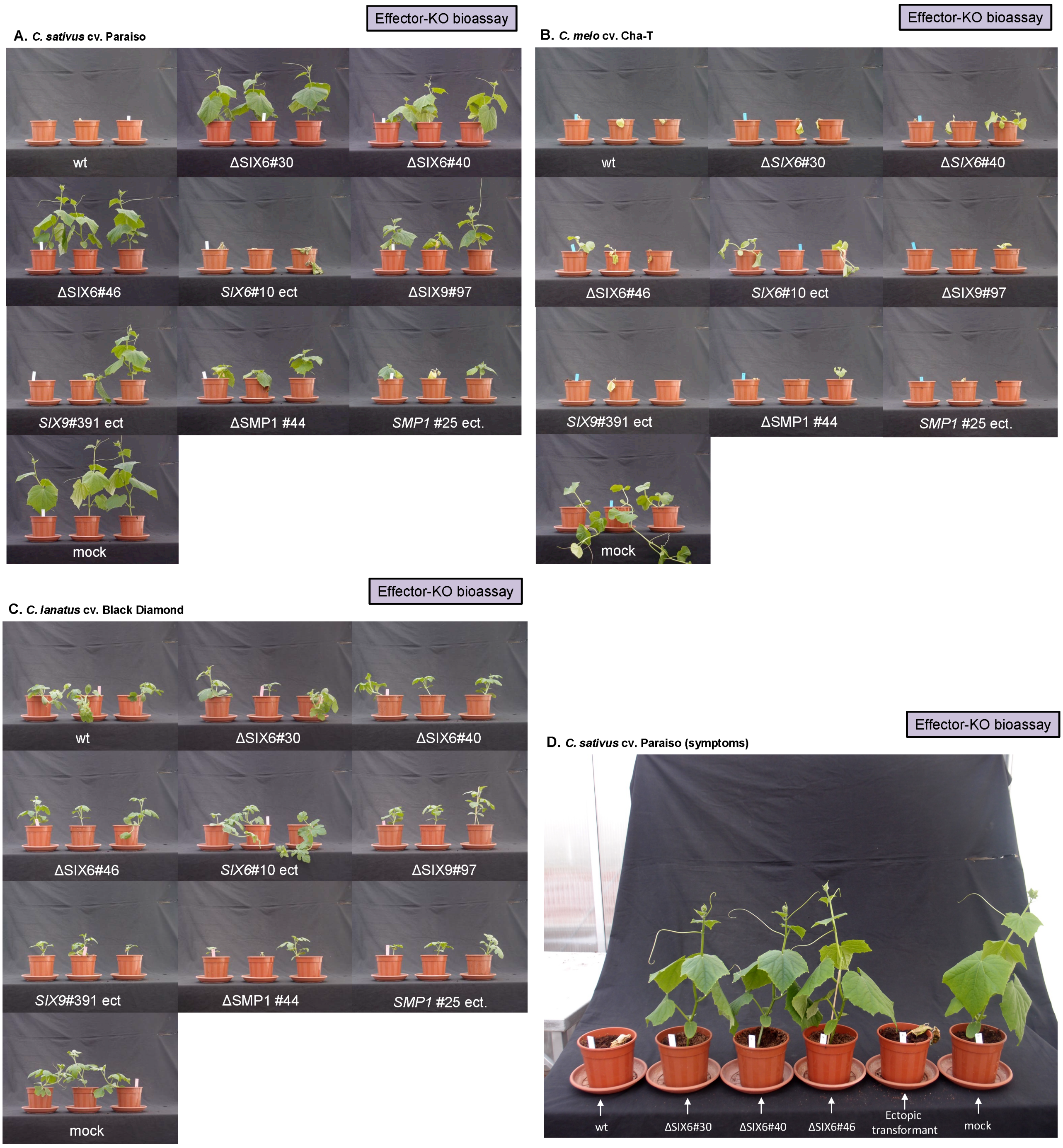

Fig S6: Symptom development in Forc016*ΔSIX6* treated plants is less strong than in the control treatments, particularly on cucumber plants.

(A) Cucumber, **(B)** melon and **(C)** watermelon plants two weeks after inoculation with different effector candidate knockout strains. The most notable difference is seen in **(D)** cucumber plants treated with three individual *SIX6* deletion strains (*ΔSIX6* #30, #40 and #46) compared to an ectopic transformant , a wildtype strain and mock.

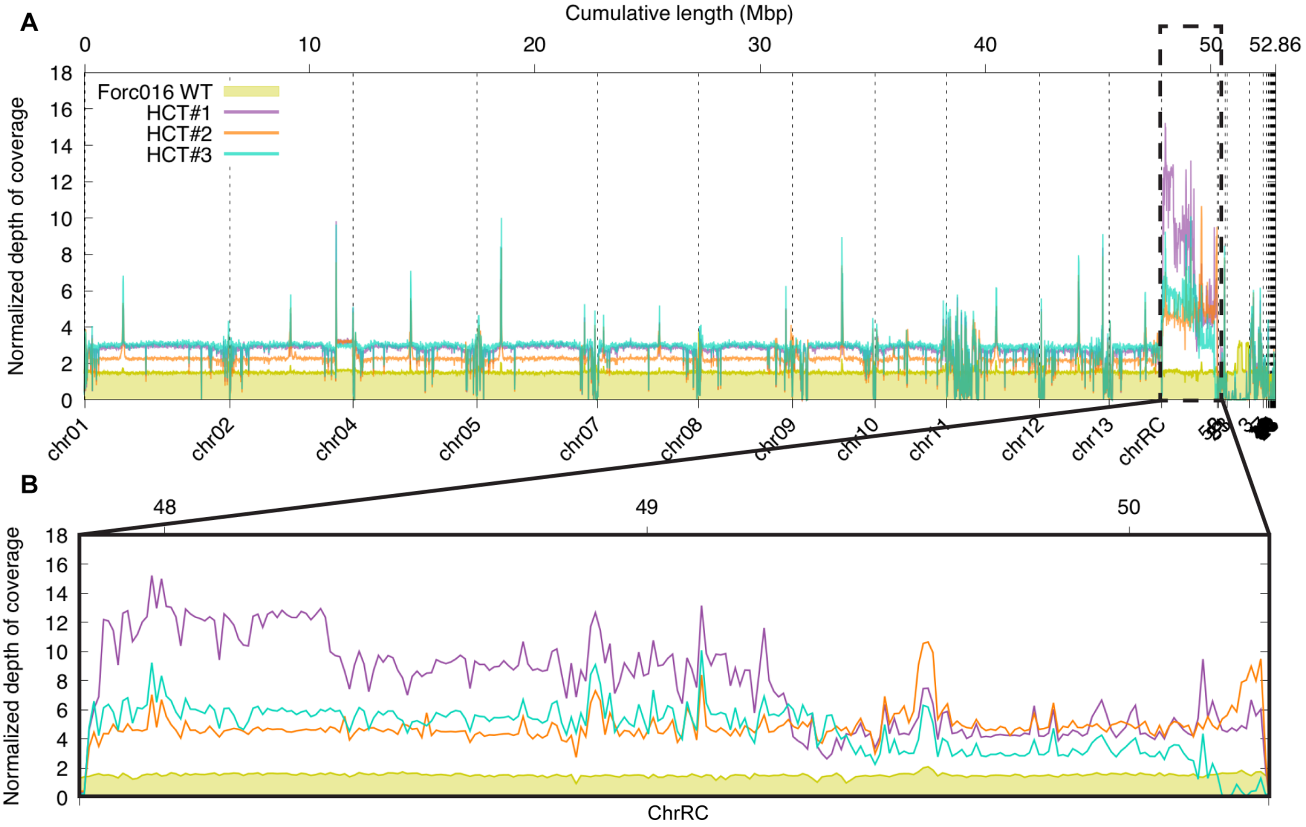

Fig S7: Normalized Illumina read mapping to the SMRT assembly of Forc016 shows large scale chromosome rearrangements and duplications upon horizontal transfer of chr^{RC} into a Fo47 background of HCT strains #1 and #3.
(**A**) Reads mapped more abundantly to the transferred chr^{RC} sequence than the rest of the assembly. **(B)** HCT strain #2 was included as a control that obtained a single copy of chr^{RC} in the Fo47 background (showing a relative coverage ±4x along the entire chromosome). HCT #1 has relative coverage depths that vary between 12x, 8x and 4x along the entire length of chr^{RC}, suggesting large segmental duplications of parts of the chromosome. HCT #3 displays coverage along the entire chromosome except for the terminal part, where the coverage drops to 0.

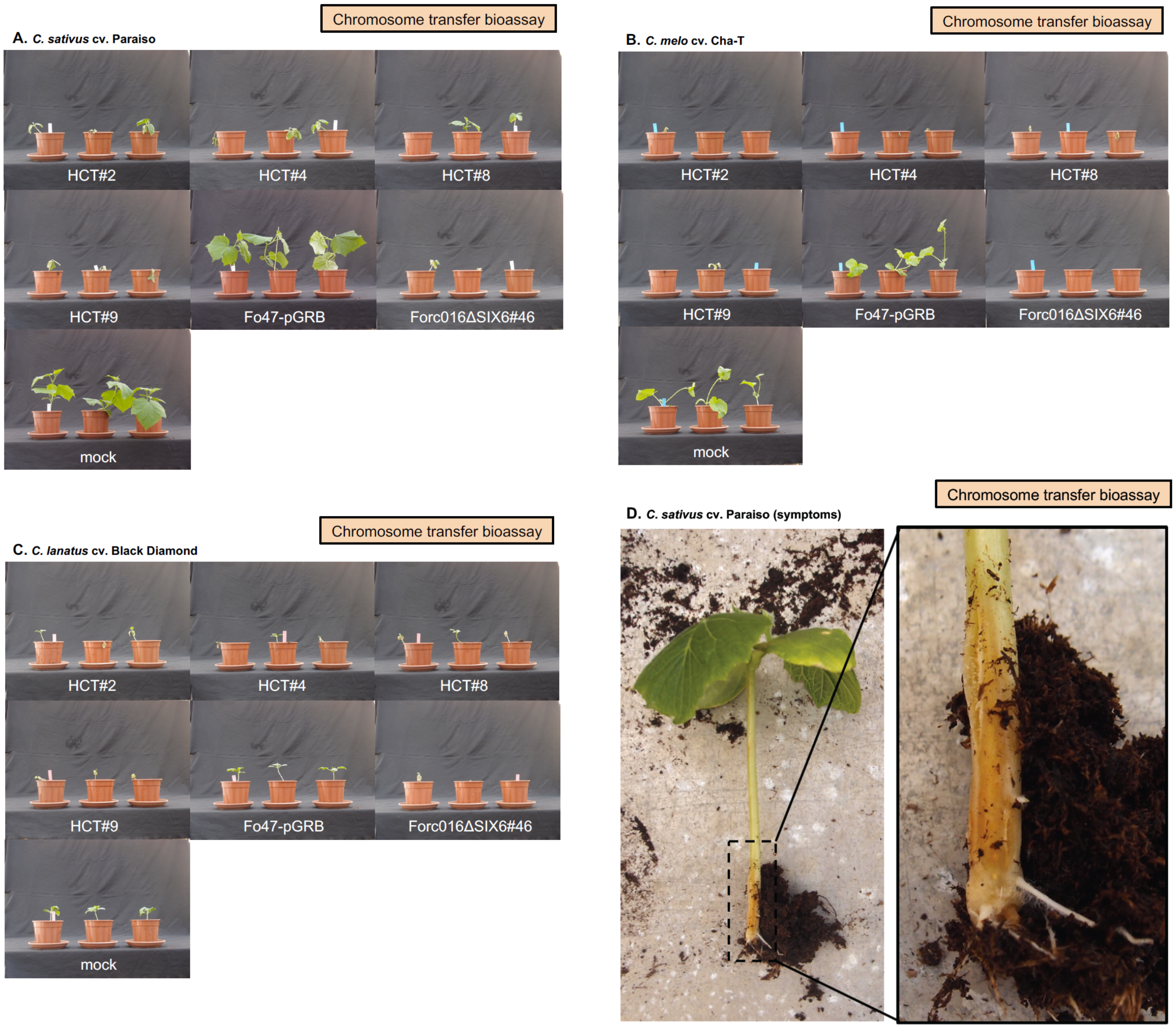

Fig S8: Horizontal chromosome transfer (HCT) of Forc chr^{RC} to Fo47 results in strains that are pathogenic on cucurbits. **(A)** Cucumber, **(B)** melon and **(C)** watermelon plants two weeks after inoculation with four HCT strains. **(D)** Typical root and shoot rot symptoms (maceration and lesion formation along the hypocotyl) associated with Forc also develop when plants are inoculated with the HCT-strains.

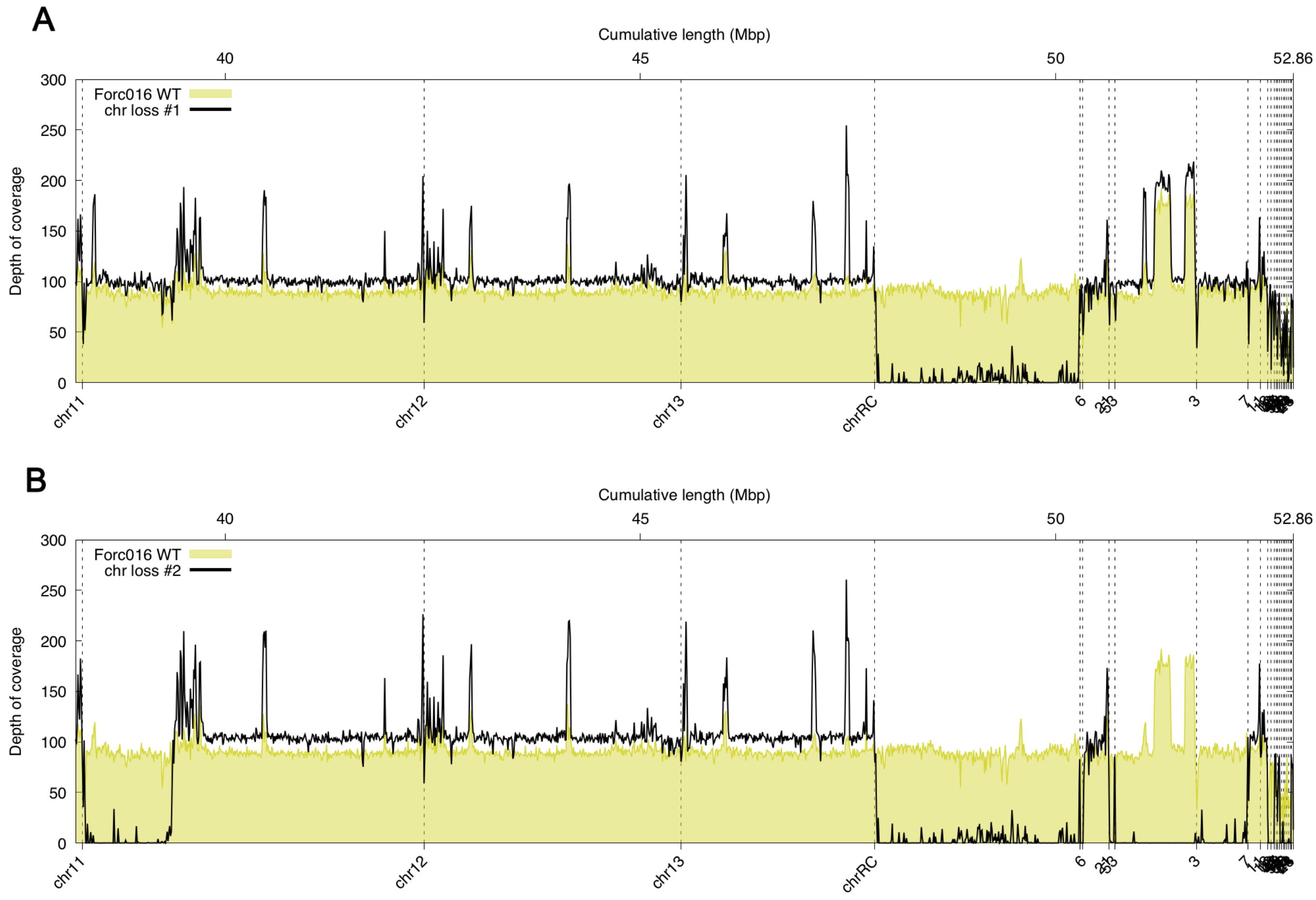

Fig S9: Illumina read mapping to the SMRT assembly of Forc016 (shown from chromosome 11 onwards) demonstrates the loss of chr^{RC} and, in strain #2, additional sequences corresponding to the two smallest chromosomes of Forc016.
(**A**) Read coverage in chr loss strain #1 indicates specific loss of chr^{RC} from the genome, while **(B)** in chr loss strain #2 this chromosome was lost along with the two small accessory chromosomes. Since no coverage was found for part of chr11 and contigs 53, 3, 21 and several smaller contigs, these together likely correspond to the two smallest chromosomes of Forc016.

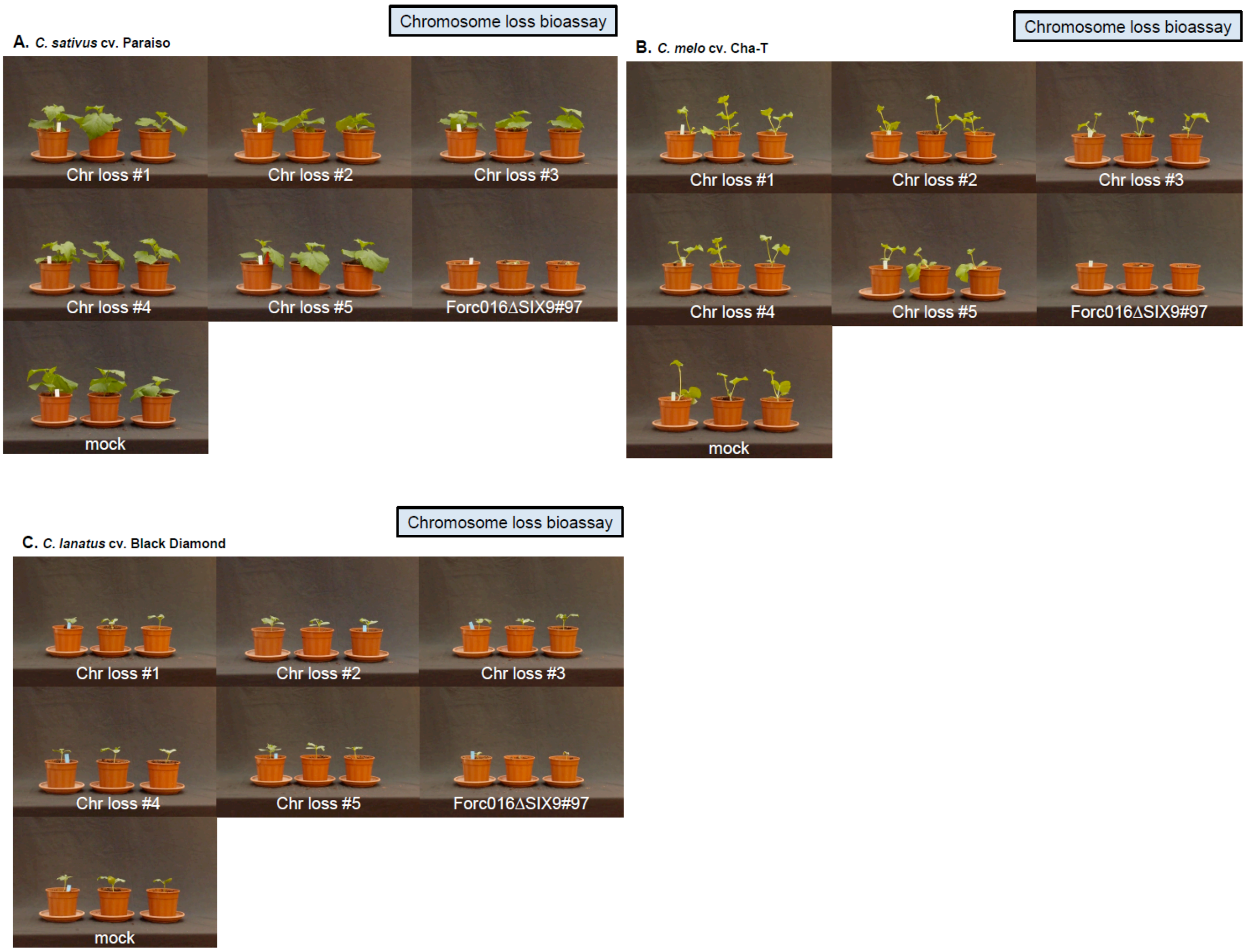

Fig S10: Forc016 strains that lost chr^{RC} have completely lost their virulence.
(A) Cucumber, **(B)** melon and **(C)** watermelon plants two weeks after inoculation with five strains that lost chr^{RC}, their parent strain (Forc016Δ*SIX9*#97) and mock clearly illustrate that only treatment with the parent strain results in disease development.

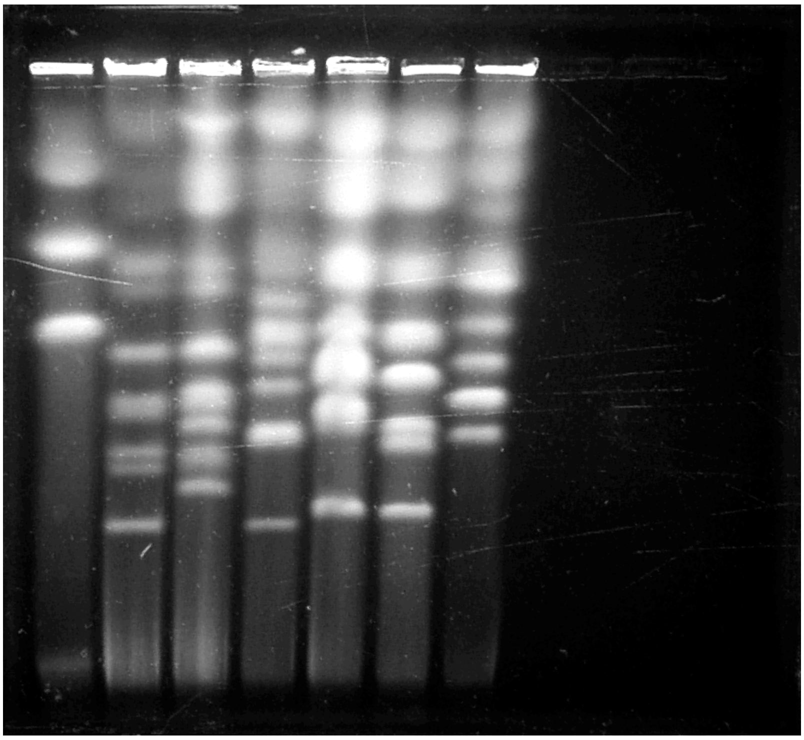

Fig S11: Uncropped CHEF gel picture of Fig 1

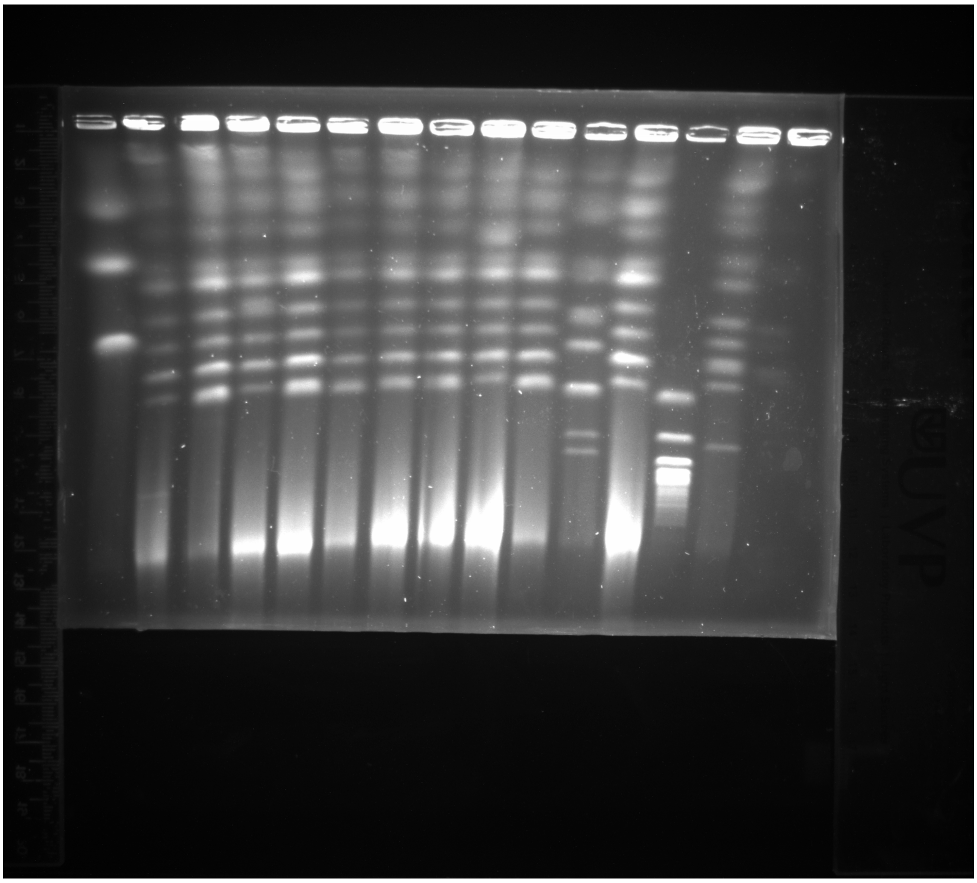

Fig S12: Uncropped CHEF gel picture of Fig 5

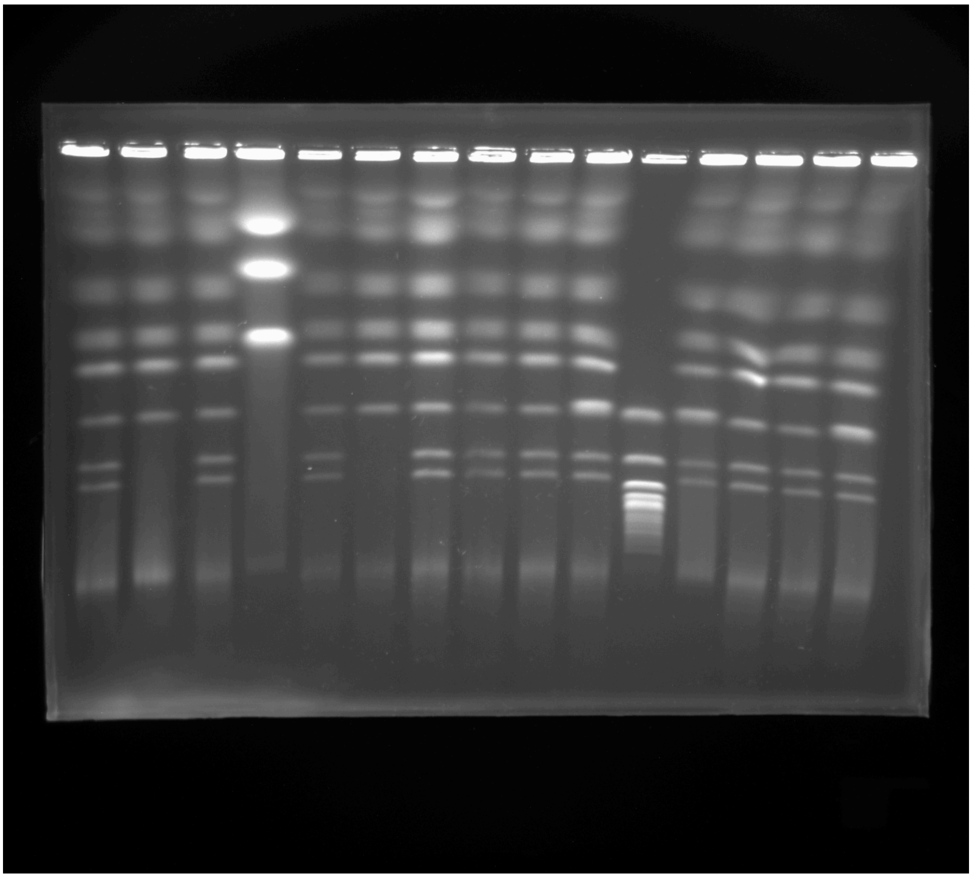

Fig S13: Uncropped CHEF gel picture of Fig 8

Table S1: Genes identified through GO term enrichment analysis (Fig S5) on chr^{RC}.

GO term	GO term name	Gene ID	Predicted function or domain	Start coordinate	End coordinate	Orien-tation
GO:0005975	carbohydrate metabolic process	g15643	Glycosyl hydrolases family 16 (GH16) domain profile	366128	367216	+
		g15837	Glycosyl hydrolase family 3 C-terminal domain	1194231	1196864	-
		g15838	Galactose mutarotase-like	1198981	1201953	+
		g15874	NodB homology domain profile	1327871	1328549	-
		g15955	Glycosyl hydrolase family 3 N terminal domain	1628362	1628772	+
GO:0005975 / GO:0006032	carbohydrate / chitin metabolic process	g15626	Chitin-binding type-1 domain profile	288485	290001	+
		g15628	Glycosyl hydrolases family 18	295552	296358	-
		g15710	Glycosyl hydrolases family 18	675899	677125	+
		g15711	Glycosyl hydrolases family 18	681983	682963	-
		g16070	Glycosyl hydrolases family 18	2106916	2107896	+
		g16071	Glycosyl hydrolases family 18	2112754	2113980	-
		g16127	Glycosyl hydrolases family 18	2384373	2385353	+
		g16128	Chitin-binding type-1 domain profile	2389593	2391109	-
GO:0006281 / GO:0000723	DNA repair / telomere maintenance	g15574	PIF1-like helicase	101447	108284	-
		g15593	Helitron helicase-like domain at N-terminus	164699	171001	-
		g15829	PIF1-like helicase	1172771	1179609	+
		g15869	PIF1-like helicase	1307727	1309605	-
		g15892	PIF1-like helicase	1382184	1389022	+
		g16110	PIF1-like helicase	2318774	2325612	-
GO:0006302	double-strand break repair	g15732	Protein involved in double-strand break repair	808370	809446	+
GO:0006464	cellular protein modification process	g15709	Tubulin-tyrosine ligase domain	669313	670359	-
		g16072	Tubulin-tyrosine ligase domain	2119755	2120552	+
GO:0006487	protein N-linked glycosylation	g15703	protein N-linked glycosylation	635263	635820	+
		g16078	protein N-linked glycosylation	2148719	2149249	-
GO:0006730	one-carbon metabolic process	g15954	Alpha-carbonic anhydrases profile	1627113	1627999	+
GO:0006816	calcium ion transport	g15679	Predicted membrane-bound protein	505326	506780	+
GO:0015074 / GO:0007059	DNA integration / chromosome	g16096	Integrase catalytic domain profile	2261720	2264886	-

	segregation					
GO:0015936	coenzyme A metabolic process	g15638	Hydroxymethylglutaryl-coenzyme A reductases family profile	332081	333238	-
		g16116	Hydroxymethylglutaryl-coenzyme A reductases family profile	2346009	2347166	+
GO:0019684	photosynthesis; light reaction	g15742	Predicted membrane-bound protein	841688	843079	+
GO:0032259	methylation	g15562	FtsJ-like methyltransferase	54702	55857	-

Table S2: Primers used in this study

**Primer**	**Target sequence**	**Primer sequence (5'-3')** [a]
FP5020	eGFP	aaaGGTACCaAGATCTaACTAGTaCTTAAGcctccggattttgagctttcg
FP4313	eGFP	aaaGGTACCgcgacacgatccagcattaatg
FP4983	HSVtk	aaaAAGCTTaCCTGCAGGaTGATCAaGGTCACCcgcggtggaattcgaattgg
FP4317	HSVtk	aaaAAGCTTgaccatgattacgccaagctcg
FP6031	*SIX6* upstream flank	aaaTTAATTAAccgaagagctggatcgtttgaag
FP6032	*SIX6* upstream flank	aaaACTAGTgatgtgacaggacaagttgatggttc
FP6033	*SIX6* downstream flank	aaaGGCGCGCCctataaagccaatacgattcgaag
FP6106	*SIX6* downstream flank	aaaCCTGCAGGgtagaataacctagatcacgtgc
FP6036	*SIX9* upstream flank	aaaTTAATTAAgtgactactgtggtccttgctg
FP6037	*SIX9* upstream flank	aaaACTAGTgactataggctgaagttagactgg
FP6038	*SIX9* downstream flank	aaaGGCGCGCCggcagagattgtccttacaaac
FP6107	*SIX9* downstream flank	aaaGGTCACCgagttttatcgcatatcatcgtcc
FP6064	*SMP1* upstream flank	aaaTTAATTAAgtaaagagaacacgttgcaaataag
FP6065	*SMP1* upstream flank	aaaACTAGTgaagtttcttgtgtaaaaagtgtgg
FP6066	*SMP1* downstream flank	aaaGGCGCGCCctggcgatgtcgaacagac
FP6115	*SMP1* downstream flank	aaaCCTGCAGGctgtcgagcaggaaaggatac
FP1490	*SIX6* southern probe	CTCTCCTGAACCATCAACTT
FP1491	*SIX6* southern probe	CAAGACCAGGTGTAGGCATT

[a] restriction sites are written in uppercase
